# Integrative Transcriptomics and Machine Learning Reveal the Association of CBX4 with Inflammation in Ulcerative Colitis as a Potential Epigenetic Regulator

**DOI:** 10.3390/biomedicines14030687

**Published:** 2026-03-17

**Authors:** Xiaohan Ma, Guangpeng Liu, Tingting Gong, Xueqi Liu

**Affiliations:** 1 Department of Gastroenterology, First Affiliated Hospital of Anhui Medical University, Hefei 230022, China; maxiaohan1988@163.com (X.M.); lgp020311@163.com (G.L.); 2 Anhui Provincial Key Laboratory of Digestive Disease, First Affiliated Hospital of Anhui Medical University, Hefei 230022, China; 3 Department of Clinical Laboratory, First Affiliated Hospital of Anhui Medical University, Hefei 230022, China; 4 Department of Nephropathy, First Affiliated Hospital of Anhui Medical University, Hefei 230022, China

**Keywords:** ulcerative colitis, epigenetic factor, machine learning, CBX4, inflammation

## Abstract

**Background/Objectives**: Epigenetic factors are increasingly recognized to contribute to the pathogenesis of intestinal diseases, yet the precise mechanisms through which these factors influence ulcerative colitis (UC) remain poorly understood. **Methods**: Transcriptome profiles pertaining to UC and genes associated with epigenetic factors (EFRGs) were retrieved from publicly accessible datasets. Candidate genes were ascertained through the intersection of differentially expressed genes (DEGs) and EFRGs. Key genes were screened through machine learning algorithms and validated via the Artificial Neural Network (ANN) model. Enrichment analysis and immune infiltration assays were conducted to elucidate the underlying mechanisms of these genes. The hub gene, CBX4 (Chromobox homolog 4), was validated through immunohistochemical analysis of both healthy controls and patients with UC, and the correlation was evaluated using UC-related clinical parameters. Additionally, CBX4 expression was knocked down in dextran sulphate sodium (DSS)-treated mice to examine its regulatory function. Unlike conventional broad-spectrum biomarker screens, this study specifically integrated epigenetic factor-related genes (EFRGs) with machine learning and experimental validation using both clinical samples and animal models. **Results**: *SMARCB1*, *JAK2*, *CBX4*, and *PPARGC1A* were identified as key genes, with SMARCB1, JAK2, and CBX4 being upregulated in the UC group, while PPARGC1A was significantly downregulated. The ANN model exhibited excellent diagnostic performance. Enrichment analysis revealed that the key genes were associated with pathways such as the “chemokine signaling pathway”. Immune cell infiltration analysis results revealed marked differences in the abundances of 13 immune cell types between the UC and control groups, and there were notable associations between immune cell infiltration and key genes. Notably, CBX4 expression was elevated in both DSS-treated mice and patients with UC, showing positive correlations with clinical indicators of UC. Further in vivo experiments revealed that silencing CBX4 alleviated DSS-induced colon damage and inflammation. **Conclusions**: This study identifies four EFRG-related key genes (SMARCB1, JAK2, CBX4, PPARGC1A) in UC, suggesting that CBX4 may play a significant role as an epigenetic regulator. CBX4 is upregulated in UC intestinal tissues, and its knockdown mitigates DSS-induced colitis. These findings provide critical theoretical support for developing targeted therapies for UC.

## 1. Introduction

Ulcerative colitis (UC) represents a chronic relapsing-remitting inflammatory bowel disease (IBD) characterized by persistent mucosal inflammation and ulcer formation in the colon and rectum [1]. It predominantly involves the colonic mucosa, with clinical manifestations including diarrhoea, abdominal pain, weight loss, and rectal bleeding. In advanced stages, UC may culminate in adverse sequelae including intestinal perforation, bleeding, and elevated susceptibility to colorectal cancer [2]. UC incidence has been rising globally, particularly across industrialized countries, with an estimated annual incidence of 2 to 14 per 100,000 individuals, and an even more elevated prevalence documented across North America, Europe, and Australia [3]. Contemporary treatment strategies encompass pharmacological therapies, surgical interventions, and dietary management. Pharmacological interventions primarily consist of anti-inflammatory drugs, namely 5-aminosalicylic acid 5-ASA and steroids, alongside immunosuppressants and biologics. While these therapies offer some efficacy, limitations remain [2,4], thereby underscoring the imperative to identify novel, highly sensitive biomarkers for UC diagnosis and treatment.

Epigenetic factors regulate gene expression without altering the intrinsic DNA sequence through regulatory mechanisms such as DNA methylation, histone modification and non-coding RNA molecules [5]. These epigenetic perturbations are pivotal to cellular processes including differentiation, growth, environmental adaptation, and aging, while also exerting crucial effects on diverse pathological conditions [6]. In recent years, research has increasingly focused on the role of epigenetic factors in disease pathogenesis. Studies have shown that DNA methylation, RNA methylation, and microRNAs contribute to the development of IBD [7]. Nevertheless, the exact contribution of epigenetic regulation to UC remains incompletely understood.

This study identified key epigenetic factor-related genes (EFRGs) in UC using transcriptomic data from public databases through bioinformatics analyses. Gene Set Enrichment Analysis (GSEA), immune infiltration analysis, and molecular regulatory network analyses were performed to unravel the functional roles of these genes. These observations provide critical implications for the development of promising diagnostic markers and clinical therapeutic protocols for UC. While numerous studies have employed machine learning for biomarker discovery in UC, our approach is distinct in several aspects. First, we focused on a specific functional gene set (EFRGs) to explore the epigenetic landscape of UC. Second, we transitioned from bioinformatic screening to functional validation in in vivo models, focusing on the specific role of CBX4 rather than providing a mere list of candidate genes. Finally, an artificial neural network (ANN) was utilized to enhance the clinical translational potential of our findings.

## 2. Materials and Methods

### 2.1. Data Collection

The GSE87473 and GSE165512 related to ulcerative colitis (UC) were gathered from the GEO database. GSE87473 (sequencing platform: GPL13158) contained colonic tissue samples from 106 UC patients and 21 controls. GSE165512 (sequencing platform: GPL16791) contained colonic tissue samples from 40 UC patients and 31 controls [8]. In total, 720 EFRGs were retrieved from the references (Table S1) [9]. Notably, the discovery dataset (GSE87473) and the validation dataset (GSE165512) were analyzed independently rather than being merged. To further ensure the robustness of our findings, an additional independent UC dataset, GSE48958 (sequencing platform: GPL6244), was retrieved from the GEO database. This dataset, which contained colonic tissue samples from 7 UC patients and 8 controls, was utilized exclusively for independent external validation of the key genes and the ANN model.

### 2.2. Differential Expression Analysis

Differentially expressed genes (DEGs) between UC and control samples from the GSE87473 dataset were ascertained using the Limma R package (v3.54.0) [10]. Screening thresholds were established as *p* < 0.05 and |log2 Fold Change| > 0.5. The top 10 up- and downregulated DEGs ranked by log2FC were showcased in a volcano plot, with their expression profiles illustrated in a heatmap.

### 2.3. Identification and Analysis of Candidate Genes

To identify candidate genes, the intersection of DEGs and EFRGs was generated with the VennDiagram R package (v 1.7.1). Subsequently, KEGG and GO analyses encompassing BPs, CCs, and MFs were conducted on candidate genes via the “clusterProfiler” R package (v 4.2.2), at a significance threshold of *p* < 0.05, to elucidate their mechanistic significance. The top 5 pathways in the aspects of BPs, CCs, MFs, and KEGG pathways that were most significantly enriched were visualized. Furthermore, candidate genes were subjected to PPI network analysis via the STRING database (confidence score ≥ 0.4). Subsequently, the candidate genes were analyzed by means of Cytoscape software (v 3.8.2). The Degree Centrality of the nodes was calculated and sorted according to the Degree Centrality values. The top 15 genes obtained were defined as candidate key genes.

### 2.4. Machine Learning for Screening Characteristic Genes

To identify key signature genes, two distinct machine learning algorithms were applied to screen candidate pivotal genes across all samples within the GSE87473 dataset. The glmnet R package (v4.1-8) was deployed to conduct LASSO regression analysis. Following 10-fold cross-validation, the model corresponding to lambda.min was designated as the optimal model to pinpoint LASSO-derived genes. In parallel, the e1071 R package (v1.7-16) was utilized to implement the SVM-RFE algorithm for evaluating feature importance. The optimal variable count was ascertained via 10-fold cross-validation and by monitoring the variation trend of RMSE values as the variable count rises, with the aim of minimizing the model’s prediction error. After that, the gene set corresponding to the lowest point of the error rate was selected as the SVM-RFE genes. Eventually, the VennDiagram R package (v1.7.1) was utilized to pinpoint the intersection of LASSO and SVM-RFE genes to delineate the signature genes. To ensure the robustness of feature selection, 10-fold cross-validation (CV) was integrated into both algorithms. In the LASSO regression, 10-fold CV was employed to determine the optimal penalty parameter (lambda.min). For SVM-RFE, we utilized the rfeControl function with 10-fold CV to evaluate model performance (Accuracy and Kappa) across various feature subsets, thereby mitigating the risk of selecting features based on random noise.

### 2.5. Key Gene Identification and Diagnostic Performance Analysis

To pinpoint key genes, the Wilcoxon test was deployed to verify the expression profiles of the signature genes across the GSE87473 and GSE165512 datasets. Characteristic genes that displayed notable UC and controls samples distinctions (*p* < 0.05) in expression levels and consistent manifestation trends in both GSE87473 and GSE165512 datasets were selected as key genes. Subsequently, to assess the discriminative capacity of key genes between UC and control samples, an ANN model was established for these genes across all samples in the GSE87473 dataset. Expression levels of the key genes were transformed into “gene scores” via min-max normalization. The “neuralnet” (v 1.44.2) and “neuralnet tools” (v 1.5.3) R packages were employed to specify 1 hidden layer and construct the ANN model. Thereafter, ROC analysis was conducted across all samples in the GSE87473 and GSE165512 datasets, and the Area Under the Curve (AUC) was calculated to assess the model’s diagnostic performance (1 > AUC > 0.7). Given the class imbalance in GSE87473 (21 controls vs. 106 UC), AUC was prioritized as the primary performance metric as it is insensitive to class distribution. The 95% confidence intervals (CI) for AUC were calculated using the bootstrap method (n = 1000). Prior to ANN construction, expression levels of the four key genes were normalized using Min–Max scaling to the range [0, 1] using the formula: xnorm = (x − xmin)/(xmax − xmin). To strictly avoid data leakage, the xmin and xmax parameters were derived exclusively from the training set (GSE87473) and subsequently applied to the external validation set (GSE165512). The ANN architecture was intentionally kept concise to prevent overfitting in a small-sample context, consisting of an input layer (4 neurons), a single hidden layer (2 neurons), and a sigmoid-activated output layer (1 neuron). To minimize the risk of overfitting, several strategies were implemented during model development: (1) Internal 5-fold cross-validation was performed within the training set to optimize hyperparameters; (2) L1 regularization (LASSO) was applied during the feature selection phase to reduce model complexity by penalizing non-essential variables; and (3) a minimalist ANN architecture with a limited number of hidden nodes was adopted to prevent the model from memorizing noise in the training data.

### 2.6. Analysis of Correlation and Chromosomal Localization

To elucidate interrelationships among key genes, the “psych” R package (v 2.1.6) was applied to determine their correlations across all samples in the GSE87473 dataset (|cor| > 0.30, *p* < 0.05). Thereafter, to delineate the chromosomal positions of key genes, the “RCircos” R package (v1.2.2) was utilized to graphically depict their chromosomal distribution.

### 2.7. GSEA and GeneMANIA Analysis

To additionally elucidate the biological roles and signaling pathways pertinent to key genes, the “psych” R package (v 2.1.6) was applied to determine correlations of key genes with other genes across all samples in the GSE87473 dataset, with correlations ranked in descending order. The “c2.cp.kegg_legacy.v2023.2.Hs.symbols.gmt” gene set was retrieved from MSigDB as the background reference set. The clusterProfiler R package (v4.2.2) was deployed for performing GSEA to enrich the ranked genes within this background set, with significance *p* < 0.05 and |normalized enrichment score (NES)| > 1. The top 10 pathways ranked by *p* from low to high were visualized. Then, to explore the interaction couplings between the key genes and other genes, an interaction network of the key genes was established via the GeneMANIA database (http://genemania.org/, accessed on 7 March 2025) to reveal the internal connections among genes.

### 2.8. Immune Infiltration Analysis

To investigate immune cell infiltration in UC and control samples, immune cell infiltration analysis was conducted in the GSE87473 dataset. The “CIBERSORT” R package (v0.1.0) was deployed to quantify infiltration scores for 22 immune cell subsets across all samples, excluding those with *p* > 0.05. Thereafter, the Wilcoxon test was deployed to compare infiltration abundances between UC and control samples, identifying immune cells with statistically significant differences (*p* < 0.05) for subsequent analysis. Subsequently, Spearman’s correlation analysis was implemented using the “psych” R package (v2.1.6) to explore intercorrelations among differentially infiltrating immune cells and between key genes and these immune cell subsets, applying significance thresholds of |cor| > 0.30 and *p* < 0.05.

### 2.9. Chemicals and Reagents

Hematoxylin–eosin (HE; Cat. No. D006-1-1) and Alcian blue–periodic acid–Schiff (AB-PAS; Cat. No. D033-1-1) staining kits were procured from Nanjing Jiancheng Bioengineering Institute (Nanjing, Jiangsu, China). Antibodies utilized for Western blot analysis comprised anti-PPARGC1A (Abcam, Cambridge, UK, ab106814), anti-JAK2 (Abcam, ab108596), anti-SMARCB1 (Cell Signaling Technology, Danvers, MA, USA, 12500S), anti-CBX4 (Proteintech, Rosemont, IL, USA, 18544-1-AP), anti-β-actin (Proteintech, 66009-1-Ig), anti-Occludin (Proteintech, 27260-1-AP), anti-E-cadherin (Cell Signaling Technology, 3195S), anti-IL-1β (Servicebio, Wuhan, China, GB11113-100), anti-IL-6 (Servicebio, GB11117-100) and anti-TNF-α (Proteintech, 17590-1-AP).

### 2.10. Human Specimen Collection

Consecutive patients with active ulcerative colitis (UC) and age- and sex-matched healthy controls were recruited at the First Affiliated Hospital of Anhui Medical University, Hefei, Anhui Province. Colonic mucosal biopsy specimens were collected and immediately fixed in 10% buffered formalin prior to subsequent immunohistochemical (IHC) staining analyses. Detailed clinical and laboratory characteristics were documented for all subjects, including age, gender, red blood cell count (RBC), white blood cell count (WBC), hemoglobin (HGB), platelet count (PLT), C-reactive protein (CRP), erythrocyte sedimentation rate (ESR), fecal calprotectin levels, Mayo clinical score, and disease extent of UC. Signed informed consent was obtained from all study participants or their legally authorized representatives. The study protocol was reviewed and sanctioned by the Ethics Committee of the First Affiliated Hospital of Anhui Medical University (approval No. PJ2024-11-55, Registration date: 20 December 2024). Detailed demographic and clinical characteristics are presented in Table 1.

### 2.11. Sample Size Calculation

In the preliminary experiment, 15 normal controls (NC) and 20 patients with ulcerative colitis (UC) were included. The sample size was calculated based on the mean and standard deviation (SD) of CBX4 immunohistochemical positivity. CBX4 expression followed a normal distribution; therefore, a two-sample *t*-test was performed using PASS software for sample size estimation. The type I error (α) was set at 0.05, and the type II error (β) was set at 0.1 (power = 90%). The mean ± SD values of CBX4 positivity were 2.695 ± 0.9493 in the healthy control group and 32.51 ± 5.285 in the UC group. Based on these parameters, the minimum required sample sizes were calculated to be 2:3 for the healthy control and UC groups, respectively. UC patients were further stratified into three subgroups: mild, moderate, and severe. The sample size was estimated based on the mean and SD of CBX4 immunohistochemical positivity. In the preliminary experiment, 6, 6, and 8 samples were included in the mild, moderate, and severe groups, respectively. Since CBX4 followed a normal distribution with homogeneity of variance, one-way analysis of variance (ANOVA) contrasts were performed using PASS 2025 software. The type I error was set at α = 0.05/3 (Bonferroni correction), and the type II error was set at β = 0.1. The mean ± SD values were 26.10 ± 2.721, 32.54 ± 2.668, and 37.30 ± 1.999 in the mild, moderate, and severe groups, respectively. Based on these calculations, the minimum required sample sizes were 8, 8, and 10 for the 3 groups. The actual sample size will be expanded as much as possible to improve statistical power.

### 2.12. Animals and Treatments

The ethical protocols for all animal studies were conducted in accordance with the guidelines approved by the Animal Research Ethics Committee of Anhui Medical University (Hefei, China). A dextran sulfate sodium (DSS)-induced colitis model was generated with 2.5% DSS (MP Biomedicals, molecular weight 35,000–50,000 Da). To explore the role of CBX4 in colitis pathogenesis, C57BL/6 mice (GemPharmatech, Nanjing, China) were randomly divided into four experimental groups: scramble-NC, sh-CBX4-NC, scramble-DSS, and sh-CBX4-DSS (Hanbio Biotechnology, Shanghai, China). CBX4 knockdown was achieved by tail vein injection of recombinant adeno-associated virus serotype 9 carrying shRNA targeting CBX4 (AAV9-sh-CBX4). Mice received 2 × 10^11^ viral genomes (vg) in 100 μL sterile PBS per mouse via tail vein injection. Control mice received the same dose of AAV9-sh-NC vector. Mice were used for subsequent experiments two weeks after virus injection to allow sufficient gene knockdown efficiency. Throughout the experimental course, body weight, stool consistency, and fecal occult blood status were evaluated daily at fixed time points. Upon experimental completion, peripheral blood samples and colon tissues were harvested for follow-up assays. Mice in the NC groups were provided with regular drinking water, while those in the DSS-treated groups received 2.5% DSS dissolved in sterile distilled water for 7 consecutive days. All animals were euthanized humanely under anesthesia before sample collection.

### 2.13. Pathological Staining and Immunohistochemistry (IHC) of Intestines

Paraffin-embedded colon tissue sections were processed for hematoxylin–eosin (HE) staining following standard protocols. After deparaffinization, antigen retrieval was conducted via high-pressure treatment in freshly prepared citrate buffer. Sections were subsequently subjected to goat serum blocking (Beyotime Biotechnology, Shanghai, China, C0265) at ambient temperature for 60 min. Following blocking solution removal, sections were incubated with primary antibodies at 4 °C overnight. Subsequently, sections were incubated with matching secondary antibodies for 1 h, and immunoreactivity was detected with diaminobenzidine (DAB). In the final experimental step, cellular nuclei were counterstained with hematoxylin, and the processed tissue sections were examined under a Zeiss light microscope (Oberkochen, Germany).

### 2.14. Western Blot

Total cellular protein was isolated from harvested colon tissue samples using RIPA lysis buffer (Beyotime Biotech Inc., Shanghai, China, P0013K) supplemented with freshly added protease and phosphatase inhibitor mixtures (Beyotime Biotech Inc., P1006 and P1081) on ice. The concentrations of the extracted total protein were determined quantitatively via the Enhanced BCA Protein Assay Kit (Beyotime Biotech Inc., P0010) in accordance with the manufacturer’s standard protocol. Equal amounts of protein lysate, normalized by the quantified concentration, were denatured by heat treatment at 100 °C for 10 min, resolved by sodium dodecyl sulfate–polyacrylamide gel electrophoresis (SDS-PAGE), and subsequently electrotransferred onto nitrocellulose membranes (Beyotime Biotech Inc., FFN08). After overnight incubation with the indicated primary antibodies at 4 °C with gentle shaking, the membranes were incubated with the Ultra High Sensitivity ECL chemiluminescence detection kit (Thermo Fisher Scientific, Waltham, MA, USA, 38554). The target protein bands were detected and visualized utilizing a professional chemiluminescence imaging and analysis system. Densitometric analysis was performed using ImageJ software (version 1.53, National Institutes of Health, Bethesda, MD, USA). Target protein intensity was normalized to the corresponding β-actin loading control from the same membrane. The normalized values were then expressed relative to the control group.

### 2.15. Statistical Analysis

Bioinformatic analyses were conducted via the R programming language (version 4.2.2) for all experimental data processing in this study. The Wilcoxon rank-sum test was utilized to evaluate intergroup differences in key experimental indices between the two independent groups. Quantitative data displayed in histogram plots were expressed as mean ± standard deviation (SD). Statistical analyses for intergroup differences were conducted via GraphPad Prism 8 software using unpaired *t*-tests or one-way analysis of variance (ANOVA). All experiments were performed independently in triplicate, with *p* < 0.05 defined as statistically significant.

## 3. Results

### 3.1. Identification and Functional Analysis of 90 Candidate Genes

**Figure 1:**
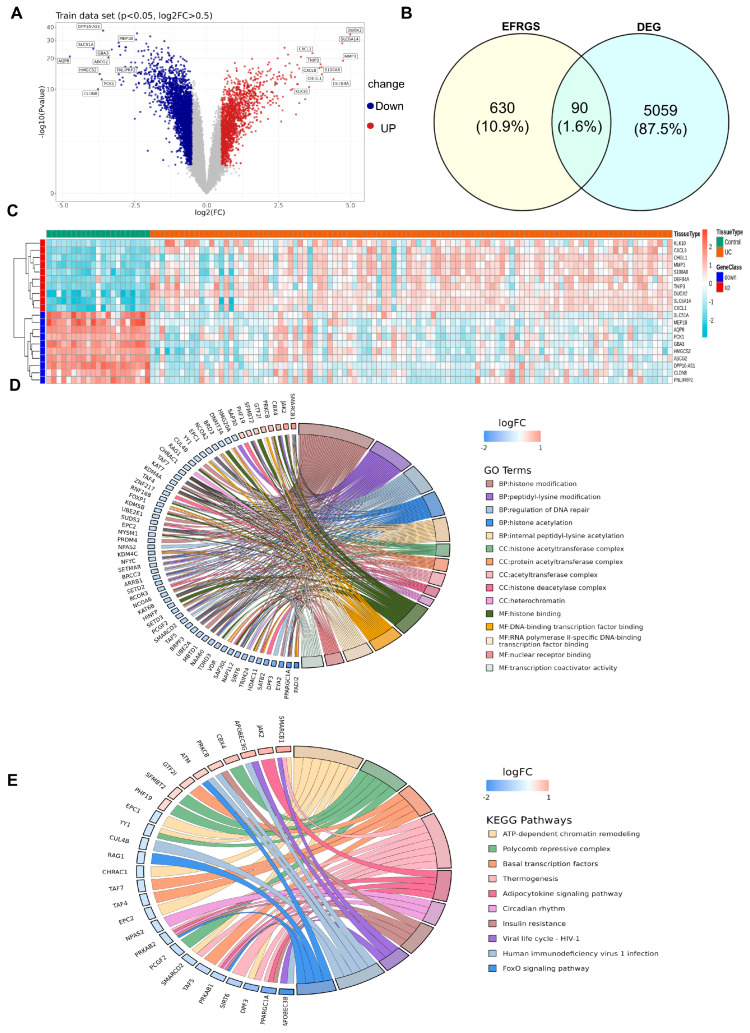
Identification and functional analysis of 90 candidate genes. (**A**) Volcano plot showed 1880 upregulated genes and 3269 downregulated genes in the UC group. (**B**) Venn plot showed a sum of 90 candidate genes obtained from the intersection between 5149 DEGs and 720 EF-RG. (**C**) Heat map. (**D**) GO enrichment analysis. (**E**) KEGG enrichment analysis.

A total of 5149 differentially expressed genes (DEGs) were detected in the GSE87473 dataset, including 1880 upregulated and 3269 downregulated transcripts (Figure 1A). Venn intersection analysis between the 720 epigenetic factors (EFRGs) and 5149 DEGs yielded 90 candidate genes (Figure 1B,C). Gene Ontology (GO) functional annotation revealed 564 enriched terms, with 407 terms related to biological processes (BPs) such as “histone modification,” 58 terms in cellular components (CCs) like “heterochromatin,” and 99 terms in molecular functions (MFs) such as “histone binding” (*p* < 0.05) (Figure 1D and Table S2). Furthermore, a total of 26 pathways from the Kyoto Encyclopedia of Genes and Genomes (KEGG) database, with the “Thermogenesis” pathway as a representative, exhibited statistically significant enrichment (*p* < 0.05) (Figure 1E and Table S3).

### 3.2. Screening of 7 Characteristic Genes SMARCB1, JAK2, CBX4, KDM4A, WHSC1, BRCC3 and PPARGC1A

**Figure 2:**
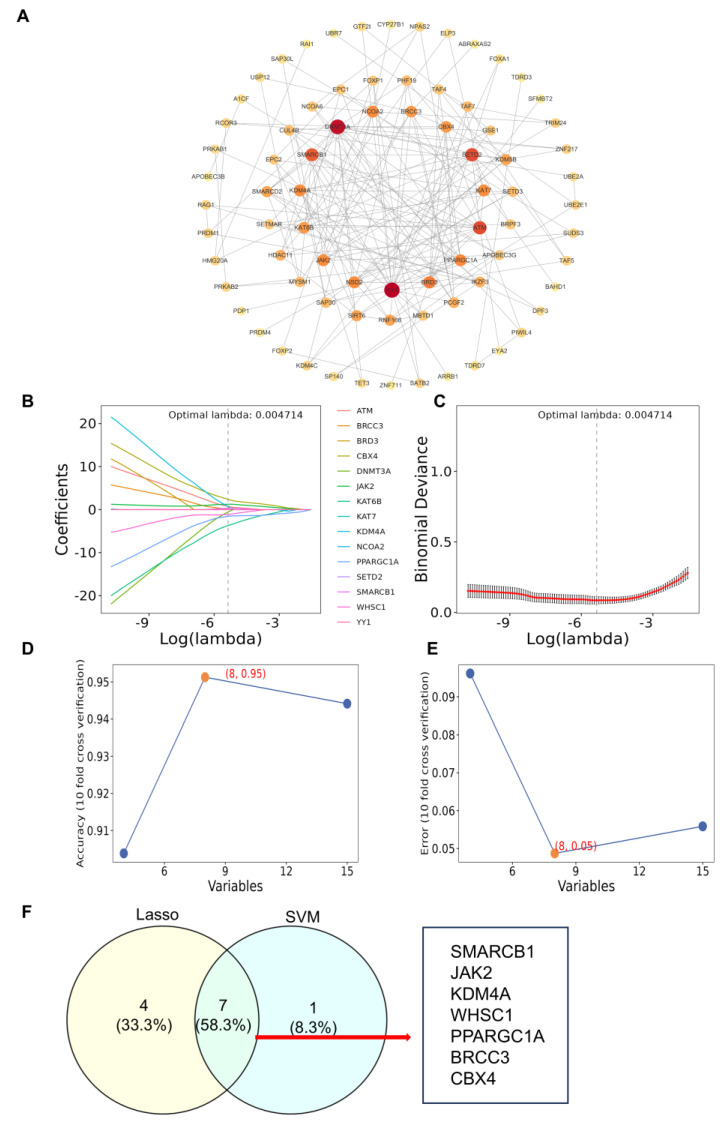
Screening of 7 characteristic genes *SMARCB1*, *JAK2*, *CBX4*, *KDM4A*, *WHSC1*, *BRCC3* and *PPARGC1A*. (**A**) PPI network. (**B**,**C**) The Lasso algorithm. (**D**,**E**) SVM-RFE algorithm. (**F**) Venn diagram based on 2 algorithms.

The protein–protein interaction PPI network of 90 candidate genes screened 15 core genes, namely *YY1*, *DNMT3A*, *ATM*, *SETD2*, *SMARCB1*, *BRD3*, *JAK2*, *KAT7*, *KDM4A*, *WHSC1*, *NCOA2*, *PPARGC1A*, *BRCC3*, *CBX4* and *KAT6B*, with 75 remaining genes showing interactions. The target genes showed the strongest binding ability (Figure 2A). LASSO regression analysis was performed, and when lambda.min was set to 0.004714, the model was optimized, yielding 11 LASSO genes (*YY1*, *DNMT3A*, *SMARCB1*, *JAK2*, *KAT7*, *KDM4A*, *WHSC1*, *NCOA2*, *PPARGC1A*, *BRCC3*, and *CBX4*) (Figure 2B,C). Concurrently, SVM-RFE analysis identified 8 genes (*PPARGC1A*, *KDM4A*, *CBX4*, *JAK2*, *WHSC1*, *SMARCB1*, *BRCC3*, and *ATM*) (Figure 2D,E). Venn analysis of the 11 LASSO genes and 8 SVM-RFE genes yielded 7 overlapping genes (*SMARCB1*, *JAK2*, *KDM4A*, *WHSC1*, *PPARGC1A*, *BRCC3*, and *CBX4*), which were defined as signature genes (Figure 2F).

### 3.3. Functional Characterization, Construction and Evaluation of ANN Model for 4 Key Genes

**Figure 3:**
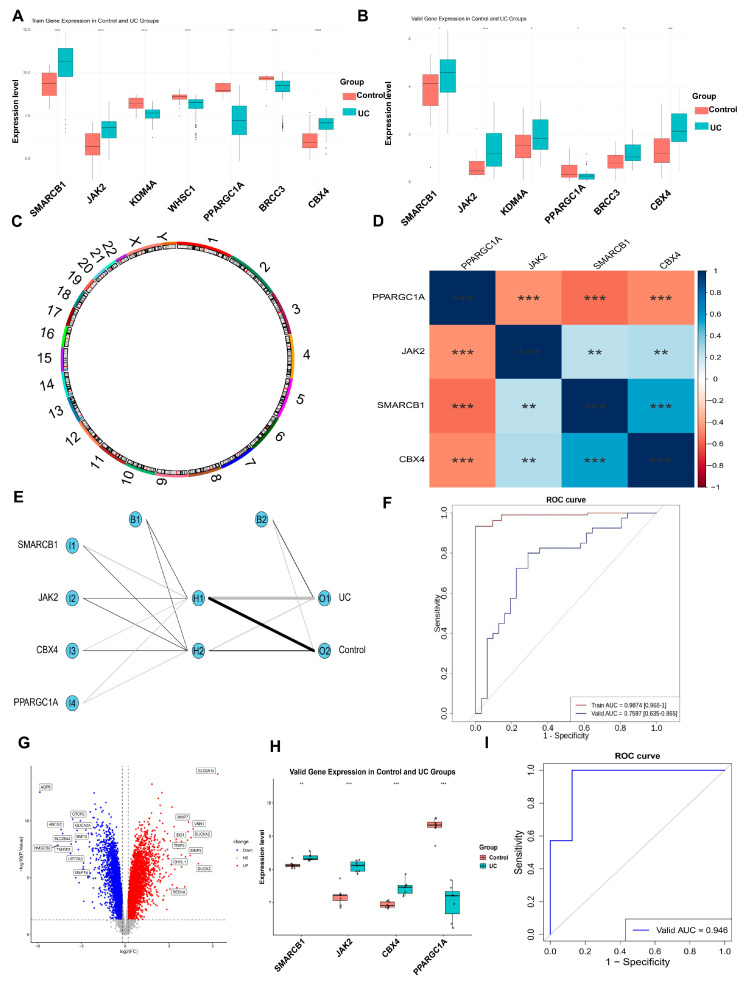
Functional characterization, construction and evaluation of ANN model for 7 key genes. (**A**,**B**) The box plot shows the differential expression between UC and the control group in the training set and the validation set. (**C**) Chromosome localization map of the key gene. (**D**) The correlation graph among the key genes. (**E**) The ANN model of the key gene. (**F**) ROC analysis was used to evaluate the diagnostic efficacy of the ANN model. (**G**) Volcano plot showed 4179 upregulated genes and 4385 downregulated genes in the UC group. (**H**) The box plot shows the differential expression between UC and the control group. (**I**) ROC curve analysis of the ANN model. * *p* < 0.05, ** *p* < 0.01, *** *p* < 0.001, **** *p* < 0.0001.

Expression analysis demonstrated differential expression of the seven signature genes across the GSE87473 and GSE165512 datasets, with consistent expression patterns noted for four genes (*SMARCB1*, *JAK2*, *CBX4*, and *PPARGC1A*; *p* < 0.05). Of these, *SMARCB1*, *JAK2*, and *CBX4* were upregulated in UC samples, whereas *PPARGC1A* was markedly downregulated. These four genes were thus defined as key genes for further analysis (Figure 3A,B). Chromosomal mapping demonstrated that *SMARCB1* resides on chromosome 22, *JAK2* on 9, *CBX4* on 17, and *PPARGC1A* on 4 (Figure 3C). Correlation analysis revealed a significant negative association between *SMARCB1* and *PPARGC1A* (r = −0.55, *p* < 0.05). Conversely, *CBX4* was positively correlated with *SMARCB1* (r = 0.56, *p* < 0.05) (Figure 3D). The Artificial Neural Network (ANN) model analysis indicated that the weight lines corresponding to the four key genes had an equal impact on the model’s output, and the structure of the network, with appropriate layers and neurons, demonstrated strong performance (Figure 3E). The area under the curve (AUC) for the ANN model was 0.9874 for the GSE87473 dataset. In the independent validation set (GSE165512), the model achieved an AUC of 0.7597 (95% CI: 0.635–0.865 (Figure 3F)), reflecting the high diagnostic accuracy of the model and the strong discriminatory power of the four key genes. The ANN model demonstrated robust performance in the training set (Accuracy: 0.953; Sensitivity: 0.972; Specificity: 0.857; PPV: 0.972; NPV: 0.857). In the external validation set (GSE165512), the model maintained high Sensitivity (1.000) and a PPV of 0.563, with an overall Accuracy of 0.563. These results provide a comprehensive evaluation of the model’s diagnostic potential. Furthermore, we validated the expression trends of the four key genes in the GSE48958 dataset (Figure 3G). Consistent with our primary findings, *SMARCB1*, *JAK2*, and *CBX4* were significantly upregulated, while *PPARGC1A* was downregulated in UC samples (Figure 3H). The ANN model also demonstrated stable diagnostic performance in this additional cohort (Figure 3I). This cross-dataset consistency underscores the reliability of our signature genes.

### 3.4. Functional Enrichment and GGI Network of Four Key Genes

**Figure 4:**
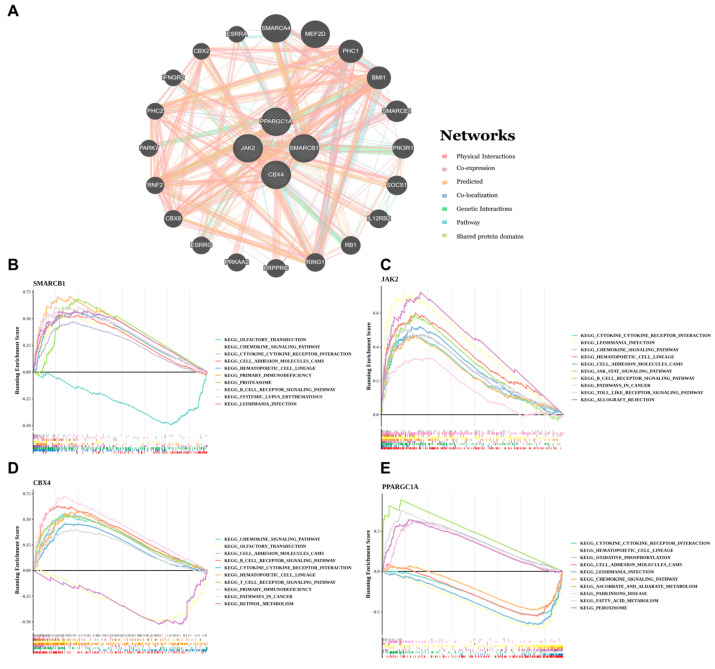
Functional enrichment and GGI network of 4 key genes. (**A**) The GeneMANIA network diagram of key genes. (**B**–**E**) GSEA enrichment analysis of key genes.

Gene–gene interaction (GGI) network analysis revealed that the four core genes exhibited interactions with 20 additional genes, and these associations mediated diverse biological functions. For instance, *SMARCB1* engaged in genetic interaction events (Figure 4A). Enrichment analysis highlighted key biologically relevant processes. Among the most significant results, we identified several core pathways including the ‘chemokine signaling pathway’ and ‘histone modification’. (Figure 4B–E). These pathways are crucial for the pathogenesis of UC (*p* < 0.05 and NES > 1).

### 3.5. Immune Infiltration Analysis of Four Key Genes

**Figure 5:**
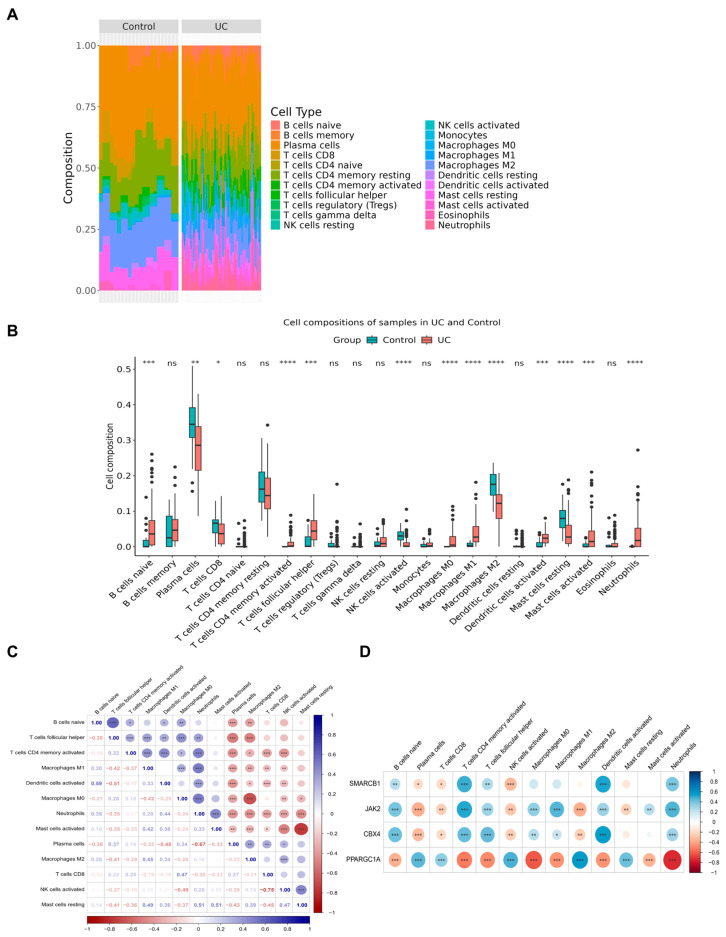
Immune infiltration analysis of 4 key genes. (**A**) The abundances of 22 immune cells in all samples of the UC group and the control group. (**B**) The differences in the infiltration of each type of immune cell in the samples of the UC group and the control group. (**C**) Correlation graph among differentially immune cells. (**D**) The correlation graph between differential immune cells and key genes. * *p* < 0.05, ** *p* < 0.01, *** *p* < 0.001, **** *p* < 0.0001, ns, not significant.

Immune function exerts a critical role in the pathogenesis of UC [11]. Of the 22 immune cell subsets, activated natural killer (NK) cells displayed the highest abundance in UC samples (Figure 5A). Comparative analysis of immune cell infiltration between UC and control samples identified marked differences in the infiltration abundance of 13 immune cell subsets, such as activated mast cells (*p* < 0.001) (Figure 5B). Correlation analysis demonstrated that CD8+ T cells exhibited the most pronounced inverse correlative relationship with activated NK cells (r = −0.75, *p* < 0.001) (Figure 5C). Furthermore, the four key genes showed significant associations with multiple immune cell populations. SMARCB1 displayed a robust correlation with activated dendritic cells, whereas JAK2 manifested a marked correlative relationship with activated CD4+ memory T cells (r = 0.52, *p* < 0.001) (Figure 5D). These findings underscore the pivotal involvement of both key genes and immune cell dynamics in UC progression.

### 3.6. CBX4 Was UpRegulated in Patients with UC

**Figure 6:**
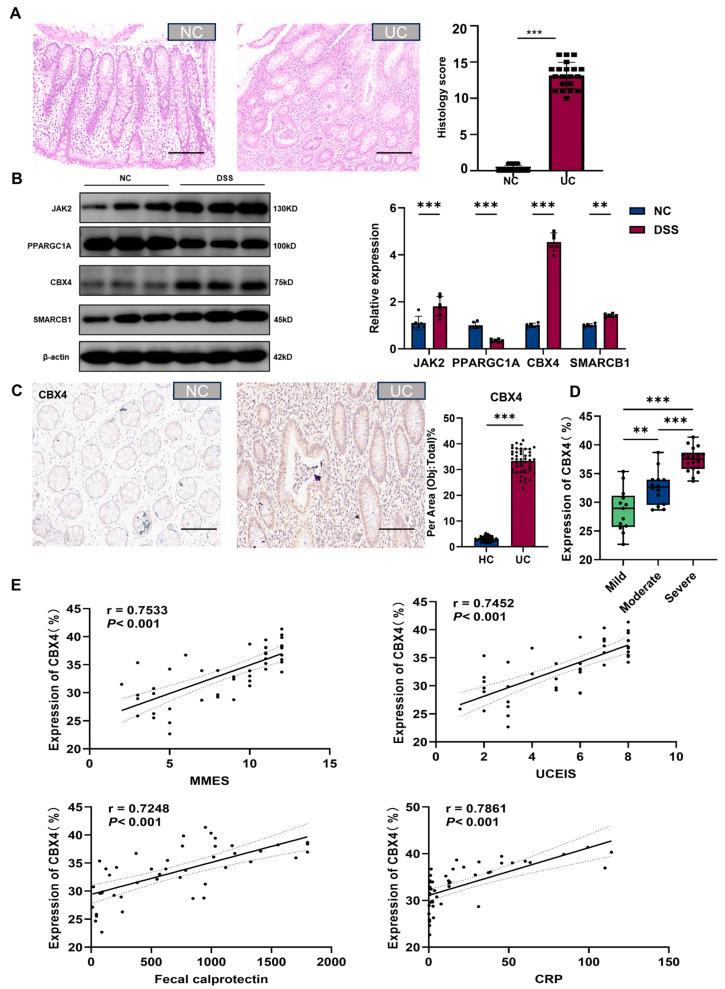
CBX4 was upregulated in UC patients. (**A**) H&E staining of NC (n = 45) and UC patients (n = 45). Scale bar = 100 μm. (**B**) Western blot analysis of 4 key genes, n = 6. (**C**,**D**) The immunohistochemical of CBX4 (n = 45). (**E**) CBX4 expression was positively correlated with multiple UC-related clinical parameters: Mayo Endoscopic Score (MMES; r = 0.7533, *p* < 0.001), ulcerative colitis endoscopic index of severity (UCEIS; r = 0.7452, *p* < 0.001), fecal calprotectin (r = 0.7248, *p* < 0.001), and C-reactive protein (CRP; r = 0.7861, *p* < 0.001). Scale bar = 50 μm. Data were presented as mean ± SD. ** *p* < 0.01, *** *p* < 0.001.

The present study enrolled 45 UC patients and 45 healthy control subjects. Hematoxylin and eosin (HE) staining showed colonic epithelial injury, crypt structural destruction, and inflammatory cell infiltration in patients with UC (Figure 6A). Western blot analysis indicated that CBX4 exhibited the most significant changes in the colon of UC mice (Figure 6B). Additionally, immunohistochemical analysis of patients with UC revealed significantly increased expression of CBX4 in their colons (Figure 6C,D). Furthermore, CBX4 expression exhibited a positive correlation with several UC-associated clinical parameters: Modified Mayo Endoscopic Score (MMES; r = 0.7533, *p* < 0.001), ulcerative colitis endoscopic index of severity (UCEIS; r = 0.7452, *p* < 0.001), fecal calprotectin (r = 0.7248, *p* < 0.001), and C-reactive protein (CRP; r = 0.7861, *p* < 0.001) (Figure 6E).

### 3.7. Silencing CBX4 Alleviates DSS-Induced UC in Mice

**Figure 7:**
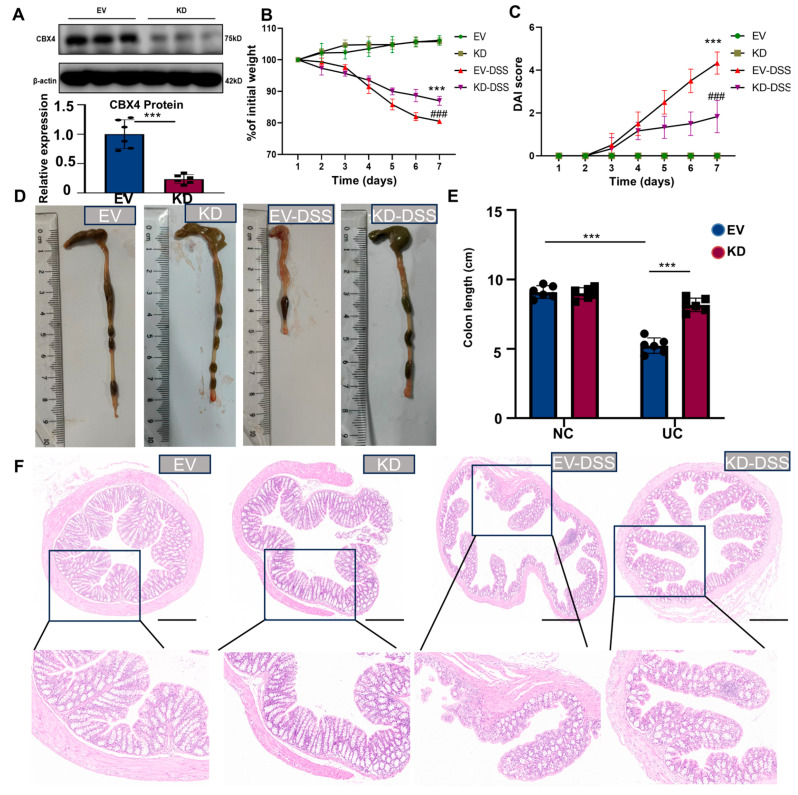
Silencing CBX4 alleviates DSS-Induced UC in Mice. (**A**) Western blot analyses showing CBX4 expression in the colons of mice following knockdown. (**B**) Body weight change in different groups. (**C**) The Disease Activity Index (DAI) in different groups. (**D**,**E**) Statistical analysis of colon length. (**F**) H&E staining. Scale bar = 100 μm. Data were presented as mean ± SD, n = 6. *** *p* < 0.001. ### *p* < 0.001 vs. EV-DSS group.

To explore the functional significance of CBX4 in a mouse model of UC, CBX4 knockdown was accomplished through tail vein delivery of the adeno-associated virus (AAV) vector AAV9-sh-CBX4. Western blot analysis verified efficient CBX4 knockdown in mouse colons (Figure 7A). Relative to control mice, DSS-induced UC mice displayed significant weight loss starting on day 4. Of note, CBX4 silencing markedly attenuated weight loss in DSS-treated animals (Figure 7B). The Disease Activity Index (DAI), derived by integrating body weight fluctuations, stool consistency, and fecal occult blood status, demonstrated a marked decrease in colitis severity among CBX4-silenced mice (Figure 7C). Moreover, DSS treatment led to a marked reduction in colon length in UC mice, whereas CBX4 knockdown effectively reversed this colon length reduction (Figure 7D,E). The HE staining results indicated that after silencing CBX4, the damage to the colonic epithelium, the destruction of crypt architecture, and the infiltration of inflammatory cells were alleviated compared to those of the DSS mice (Figure 7F).

### 3.8. Silencing CBX4 Ameliorated Intestinal Inflammatory Responses and Mucosal Barrier Function in UC Mice

**Figure 8:**
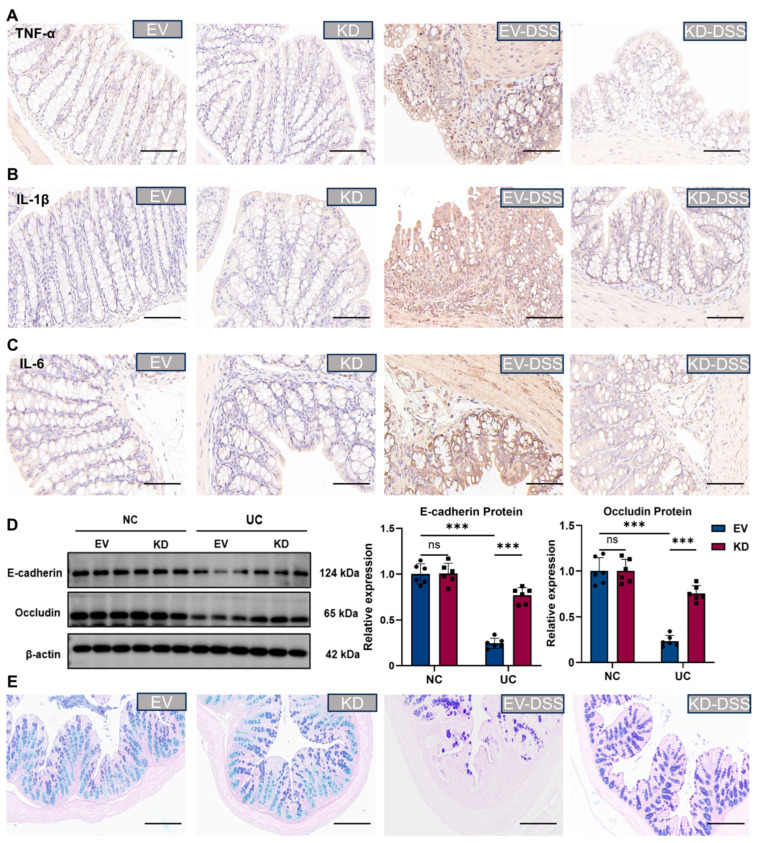
Silencing CBX4 ameliorated intestinal inflammatory responses and mucosal barrier function in UC mice. (**A**–**C**) Immunohistochemistry of inflammatory factors TNF-α, IL-1β, and IL-6. Scale bar = 50 μm. (**D**) Western blot. (**E**) Representative images of AB-PAS staining. Scale bar = 50 μm. Data were presented as mean ± SD, n = 6. *** *p* < 0.001, ns, not significant.

Immunohistochemical staining revealed that, relative to the control group, the levels of inflammatory factors TNF-α, IL-1β, and IL-6 were markedly upregulated in the DSS group, whereas CBX4 silencing mitigated this upregulation (Figure 8A–C). To elucidate the impact of CBX4 on the intestinal epithelial barrier in DSS-induced colitis mice, the expression of tight junction and adherens junction proteins was assessed by Western blotting, which demonstrated that CBX4 knockdown rescued the expression of E-cadherin and Occludin (Figure 8D). The results of the AB-PAS staining indicated that the mucin in the intestinal tissues of mice in the DSS group was significantly reduced compared with that in the NC group, indicating that the mucus barrier was damaged. However, silencing CBX4 significantly alleviated this symptom (Figure 8E).

## 4. Discussion

The onset of UC is closely associated with both genetic and environmental factors. Its pathogenesis primarily involves immune responses and inflammation in the intestinal mucosa; however, the specific mechanisms and pathogenic factors remain unclear [12]. Epigenetics serves as a bridge for exploring the interaction between genetic and environmental influences with significant clinical implications as both a diagnostic biomarker and a target for therapeutic intervention [13,14,15]. In this study, transcriptomic data from UC and EFRGs were sourced from public databases. Key genes, including *SMARCB1*, *JAK2*, *CBX4*, and *PPARGC1A*, were identified. The constructed ANN model exhibited excellent diagnostic performance. Enrichment analysis revealed that these key genes were associated with pathways such as the “chemokine signalling pathway.” Furthermore, the infiltration of 13 immune cell types, including M0 macrophages, showed significant differences between UC and control samples, with immune cell infiltration strongly correlating with the key genes. Notably, CBX4 was highly expressed in DSS-treated mice and patients with UC, exhibiting positive correlations with clinical indicators of UC. In vivo experiments demonstrated that silencing CBX4 mitigated DSS-induced colon damage and inflammation.

The strategy of using a discovery cohort followed by independent validation aligns with the methodology demonstrated by Song et al. [16], who emphasized the significance of cross-cohort validation for robust biomarker identification. Furthermore, as single-cell sequencing technologies become more prevalent, future efforts to extend these findings to the single-cell level could benefit from computational frameworks like scLM, developed by Zhang et al. [17]. Such methods can effectively handle technical heterogeneity and batch effects across multiple datasets, enabling a more granular resolution of the functional roles of genes like *CBX4*. The identification of *SMARCB1* and *CBX4*—genes rarely reported in previous UC studies—highlights the efficacy of our integrative EFRG-based approach combined with machine learning. While datasets like GSE165512 have previously yielded candidates such as *AQP9* and *IFITM1* using traditional differential expression analysis, our focus on epigenetic factor-related genes allowed us to uncover these novel regulators [18]. The consistent validation across three independent datasets (GSE87473, GSE165512, and GSE48958) suggests that CBX4 and SMARCB1 are robust biological markers of UC inflammation, rather than cohort-specific noise.

CBX4 binds to specific chromatin regions via its chromatin-binding domains, contributing to the formation and maintenance of heterochromatin and playing a pivotal role in gene silencing and the organization of higher-level chromatin structures [19]. It also functions as a transcriptional regulator, precisely modulating gene expression [20]. CBX4 is implicated in the initiation and progression of various cancers. In tumors such as breast, lung, and colorectal cancers, CBX4 is highly expressed and may promote tumor growth and metastasis by mechanisms including the stimulation of cell proliferation, inhibition of apoptosis, and enhancement of cell migration and invasion [21]. Additionally, CBX4 is essential for the development and functional maintenance of the nervous system. Abnormal expression or dysfunction of CBX4 has been linked to pathological changes in nerve cells [22]. However, the role of CBX4 in UC has not been reported so far. Previous studies have shown that CBX4 can regulate inflammation and epithelial barrier disruption in asthma [23]. Moreover, CBX4 participates in the SUMO modification of BRD4, influencing its ability to regulate the expression of pro-inflammatory genes such as IL-1β, TNF-α, and IL-6 in synovial fibroblasts [24]. The role of CBX4 in UC, however, remains unreported. CBX4 was highly expressed in DSS-treated mice and patients with UC, with positive correlations to clinical indicators of UC. Furthermore, in vivo experiments demonstrated that silencing CBX4 alleviated DSS-induced colon damage and inflammation.

This study also revealed that SMARCB1 and JAK2 were upregulated in the UC group, while PPARGC1A was notably downregulated. JAK2 is broadly expressed across various tissues and cell types, including hematopoietic cells, lymphocytes, endothelial cells, and fibroblasts [25]. As a key component of the JAK-STAT signaling pathway [26], JAK2’s abnormal activation is implicated in the pathogenesis of autoimmune diseases such as rheumatoid arthritis and IBD [27]. Inhibiting the JAK-STAT pathway can alleviate UC symptoms [26]. PPARGC1A, on the other hand, is predominantly expressed in tissues with high energy demands, such as brown adipose tissue, skeletal muscle, liver, and heart [28]. It plays a pivotal role in regulating mitochondrial biogenesis, promoting mitochondrial generation and maintenance, which enhances cellular energy metabolism [29]. PPARGC1A also induces the expression of antioxidant enzymes, bolstering cellular antioxidant defenses and reducing oxidative stress [30]. Abnormal expression or mutations in PPARGC1A can lead to insulin resistance and disruptions in glucose and lipid metabolism [31]. SMARCB1 is involved in chromatin remodeling [32] and modification by interacting with chromatin-associated proteins, influencing the structure and function of chromatin [33]. In this study, SMARCB1 and JAK2 were upregulated in the UC group, while PPARGC1A expression was significantly reduced.

The cytokine–cytokine receptor interaction signaling pathway, which involves various cytokines and their receptors, plays a critical role in UC [34,35]. Analysis of patients with UC revealed that key activated pathways included cytokine–cytokine receptor interactions, the chemokine signaling pathway, and complement and coagulation cascades [36]. Additionally, patients with UC who were primarily unresponsive to infliximab treatment exhibited enrichment in cytokine–cytokine receptor interactions and IL-17 signaling pathways [37]. In this study, the cytokine–cytokine receptor interaction signaling pathway was found to be significantly enriched in the four key genes (*SMARCB1*, *JAK2*, *CBX4*, *PPARGC1A*), suggesting their pivotal roles in the development of UC.

Memory T cells (mTcells) and memory T follicular cells (mTfh) play critical roles in the pathogenesis of IBD [38]. During the initial immune response, T cells are activated and differentiate into effector T cells. Following antigen binding and elimination, most effector T cells undergo apoptosis, while a subset becomes memory T cells [39]. These memory cells maintain immune homeostasis and ensure immune balance by rapidly responding to secondary antigen exposure [40]. However, if the function of memory T cells is dysregulated, secondary antigen stimulation can lead to the differentiation of memory T cells into aberrant effector T cells, which may contribute to the development of autoimmune diseases such as UC and Crohn’s disease (CD) [40]. This study also highlighted the significant correlation between the immune microenvironment in UC, particularly activated CD4 memory T cells, and the four key genes.

UC, a form of IBD, is characterized by widespread inflammation and ulceration of the colon and rectal mucosa, which can extend to the cecum [12]. Epigenetic modifications, such as DNA methylation, histone modifications, and non-coding RNAs, are involved in the differentiation, maturation, and functional modulation of various immune and non-immune cells [41,42]. These modifications are altered in several chronic inflammatory diseases, including UC [5]. In this study, silencing CBX4 was found to ameliorate DSS-induced colon damage and inflammation, providing further insight into its potential role in UC pathology.

Limitations of this study should be acknowledged. First, the sample size for clinical validation and the depth of mechanistic exploration remain relatively limited. While we demonstrated that CBX4 knockdown alleviates DSS-induced colitis, the precise molecular pathways through which CBX4 regulates the epigenetic landscape in UC require further investigation using techniques such as ChIP-seq or ATAC-seq. Future studies with larger cohorts and more detailed molecular assays are warranted to fully elucidate its central regulatory role. Second, in this study, independent datasets from different platforms (GPL13158 for GSE87473, GPL16791 for GSE165512 and GPL17467 for GSE48985) were utilized for external validation. Although technical variations between platforms, such as probe design and detection sensitivity, might influence the comparability of absolute gene expression levels, the four key genes (*SMARCB1*, *JAK2*, *CBX4*, and *PPARGC1A*) exhibited highly consistent expression trends across the three cohorts. This consistency across different sequencing platforms underscores the reproducibility and robustness of our findings. Although CBX4 knockdown experiments demonstrated a functional role of CBX4 in colitis progression, gain-of-function or rescue experiments were not performed in the present study. Further studies will be required to comprehensively validate the regulatory role of CBX4 in UC.

Potential overfitting in the machine learning model should be noted. The ANN model achieved a higher AUC in the discovery set (0.9874) compared to the external validation set (0.7597). Several factors may contribute to this discrepancy: (1) technical heterogeneity between sequencing platforms (GPL13158 vs. GPL16791), which introduces batch effects that are difficult to fully eliminate in cross-platform validation; (2) limited sample size in the discovery cohort (106 UC vs. 21 controls), which may lead the model to over-capture features specific to that dataset; and (3) biological heterogeneity of UC across different patient populations. Despite the performance drop, an AUC of 0.7597 still indicates that the four key genes possess significant discriminative power across independent cohorts. Future studies with multi-center, larger-scale data and more robust training strategies (e.g., stricter regularization) are required to further enhance model generalization. It is important to note that our enrichment and immune infiltration analyses serve as an exploratory screening to generate biological hypotheses. While we focused on the most statistically significant and biologically plausible pathways, future experimental studies are required to confirm these specific molecular interactions.

## 5. Conclusions

Integrated bioinformatics analyses identified *SMARCB1*, *JAK2*, *CBX4*, and *PPARGC1A* as hub genes. The established artificial neural network (ANN) model exhibited robust diagnostic efficacy, and functional enrichment analysis linked these hub genes to the chemokine signaling pathway. Moreover, the infiltration of 13 immune cell types differed significantly between the UC and control groups, and their abundance was strongly correlated with the key genes. Of note, the hub gene CBX4 was overexpressed in the colonic tissues of UC patients, and silencing CBX4 mitigated intestinal inflammation in DSS-induced colitis. Our results provide a novel mechanistic basis for developing therapeutic strategies against UC.

## Figures and Tables

**Table 1:** Clinical characteristics of included population.

Characteristic	Healthy Control Subjects (n = 45)	Patients with Active UC (n = 45)	*p* Value
Age [mean(SD), yr]	46.22 ± 1.830	42.27 ± 2.120	0.3318
Sex (female: male)	17/28	19/26	0.9026
BMI [mean(SD), kg/m^2^]	23.09 ± 0.4752	21.86 ± 0.4044	0.2883
Disease Location, n (%)			
Ulcerative proctitis (E1)		13 (32.5)	
Left-sided colitis (E2)		18 (45)	
Extensive colitis (E3)		14 (35)	
Severity of UC, n (%)			
Mild		13 (28.9)	
Moderate		14 (31.1)	
Severe		18 (40)	
MMES score		8.42 ± 0.4969	
UCEIS score		5.42 ± 0.3356	
CRP (mg/L)		22.20 ± 4.611	
ESR (mm/h)		28.09 ± 3.669	
Fecal calprotectin (μg/g)		690.80 ± 82.62	
WBC (10^9^/L)	5.893 ± 0.1984	7.88 ± 0.3921	<0.0001
RBC (10^9^/L)	4.597 ± 0.0704	4.2 ± 0.1405	<0.0001
PLT (10^9^/L)	214.6 ± 8.510	288.8 ± 18.63	<0.0001
HGB (g/L)	137.5 ± 2.277	115.1 ± 4.016	0.0003
ALB (g/L)	42.98 ± 0.4742	37.41 ± 0.7835	0.0009

## Data Availability

The data are available within the article and its Supplementary Materials.

## Supplementary Materials

The following supporting information can be downloaded at: https://www.mdpi.com/article/10.3390/biomedicines14030687/s1. Table S1: Epigenetic factor-related genes; Table S2: Gene Ontolog functional annotation; Table S3: Kyoto Encyclopedia of Genes and Genomes database.

## Abbreviations

The following abbreviations are used in this manuscript:

5-ASA	5-aminosalicylic acid
ANN	Artificial Neural Network
AUC	Area Under the Curve
BPs	Biological Processes
CBX4	Chromobox Homolog 4
CCs	Cellular Components
CRP	C-reactive Protein
DEGs	Differentially Expressed Genes
DSS	Dextran Sulfate Sodium Salt
EFRGs	Epigenetic Factor-related Genes
ESR	Erythrocyte Sedimentation Rate
GGI	Gene–Gene Interaction
GO	Gene Ontology
GSEA	Gene Set Enrichment Analysis
HGB	Hemoglobin
IBD	Inflammatory Bowel Disease
KEGG	Kyoto Encyclopedia of Genes and Genomes
MFs	Molecular Functions
PLT	Platelets
RBC	Red Blood Cell
PPI	The Protein–Protein Interaction
UC	Ulcerative Colitis
WBC	White Blood Cell
