# Supplementary material for: Integrative Transcriptomics and Machine Learning Reveal the Association of CBX4 with Inflammation in Ulcerative Colitis as a Potential Epigenetic Regulator
Source: Biomedicines. 2026 Mar 17;14(3):687. doi: 10.3390/biomedicines14030687 (PMC13023902; doi:10.3390/biomedicines14030687)
Supplement: Supplementary file 1 [file biomedicines-14-00687-s001.zip › biomedicines-4154838-supplementary.pdf]

Table S1

gene_symbol
A1CF
ACTB
ACTL6A
ACTL6B
ACTR3B
ACTR5
ACTR6
ACTR8
ADNP
AEBP2
AICDA
AIRE
ALKBH1
ANKRD32
ANP32A
ANP32B
ANP32E
APBB1
APEX1
APOBEC1
APOBEC2
APOBEC3A
APOBEC3B
APOBEC3C
APOBEC3D
APOBEC3F
APOBEC3G
APOBEC3H
ARID1A
ARID1B
ARID2
ARID4A
ARID4B
ARNTL
ARRB1
ASF1A
ASF1B
ASH1L
ASH2L
ASXL1
ASXL2

ASXL3
ATAD2
ATAD2B
ATF2
ATF7IP
ATM
ATN1
ATR
ATRX
ATXN7
ATXN7L3
AURKA
AURKB
AURKC
BABAM1
BAHD1
BANP
BAP1
BARD1
BAZ1A
BAZ1B
BAZ2A
BAZ2B
BCOR
BCORL1
BMI1
BPTF
BRCA1
BRCA2
BRCC3
BRD1
BRD2
BRD3
BRD4
BRD7
BRD8
BRD9
BRDT
BRE
BRMS1
BRMS1L
BRPF1
BRPF3
BRWD1

BRWD3
BUB1
C11orf30
C14orf169
C17orf49
CARM1
CBX1
CBX2
CBX3
CBX4
CBX5
CBX6
CBX7
CBX8
CCDC101
CDC6
CDC73
CDK1
CDK17
CDK2
CDK3
CDK5
CDK7
CDK9
CDY1
CDY1B
CDY2A
CDY2B
CDYL
CDYL2
CECR2
CENPC
CHAF1A
CHAF1B
CHD1
CHD1L
CHD2
CHD3
CHD4
CHD5
CHD6
CHD7
CHD8
CHD9

CHEK1
CHRAC1
CHTOP
CHUK
CIR1
CIT
CLNS1A
CLOCK
CRB2
CREBBP
CSNK2A1
CSRP2BP
CTBP1
CTBP2
CTCF
CTCFL
CTR9
CUL1
CUL2
CUL3
CUL4A
CUL4B
CUL5
CXXC1
DAPK3
DAXX
DDB1
DDB2
DDX21
DDX50
DEK
DMAP1
DNAJC1
DNAJC2
DND1
DNMT1
DNMT3A
DNMT3B
DNMT3L
DNTTIP2
DOT1L
DPF1
DPF2
DPF3

DPPA3
DPY30
DR1
DTX3L
DZIP3
E2F6
EED
EHMT1
EHMT2
EID1
EID2
EID2B
ELP2
ELP3
ELP4
ELP5
ELP6
ENY2
EP300
EP400
EPC1
EPC2
ERBB4
ERCC6
EXOSC1
EXOSC2
EXOSC3
EXOSC4
EXOSC5
EXOSC6
EXOSC7
EXOSC8
EXOSC9
EYA1
EYA2
EYA3
EYA4
EZH1
EZH2
FAM175A
FAM175B
FBL
FBRS
FBRSL1

FOXA1
FOXO1
FOXP1
FOXP2
FOXP3
FOXP4
GADD45A
GADD45B
GADD45G
GATAD1
GATAD2A
GATAD2B
GFI1
GFI1B
GLYR1
GSE1
GSG2
GTF2I
GTF3C4
HAT1
HCFC1
HCFC2
HDAC1
HDAC10
HDAC11
HDAC2
HDAC3
HDAC4
HDAC5
HDAC6
HDAC7
HDAC8
HDAC9
HDGF
HDGFL2
HELLS
HIF1AN
HINFP
HIRA
HIRIP3
HJURP
HLCS
HLTF
HMG20A

HMG20B
HMGB1
HMGN1
HMGN2
HMGN3
HMGN4
HMGN5
HP1BP3
HR
HSPA1A
HSPA1B
HUWE1
IKBKAP
IKZF1
IKZF3
ING1
ING2
ING3
ING4
ING5
INO80
INO80B
INO80C
INO80D
INO80E
JADE1
JADE2
JADE3
JAK2
JARID2
JDP2
JMJD1C
JMJD6
KANSL1
KANSL2
KANSL3
KAT2A
KAT2B
KAT5
KAT6A
KAT6B
KAT7
KAT8
KDM1A

KDM1B
KDM2A
KDM2B
KDM3A
KDM3B
KDM4A
KDM4B
KDM4C
KDM4D
KDM4E
KDM5A
KDM5B
KDM5C
KDM5D
KDM6A
KDM6B
KDM7A
KDM8
KEAP1
KMT2A
KMT2B
KMT2C
KMT2D
KMT2E
L3MBTL1
L3MBTL2
L3MBTL3
L3MBTL4
LAS1L
LBR
LEO1
LRWD1
MAP3K7
MAPKAPK3
MASTL
MAX
MAZ
MBD1
MBD2
MBD3
MBD4
MBD5
MBD6
MBIP

MBTD1
MCRS1
MDC1
MEAF6
MECP2
MEN1
MGA
MGEA5
MINA
MLLT1
MLLT10
MLLT6
MORF4L1
MORF4L2
MOV10
MPHOSPH8
MRGBP
MSH6
MSL1
MSL2
MSL3
MST1
MTA1
MTA2
MTA3
MTF2
MUM1
MYBBP1A
MYO1C
MYSM1
NAA60
NAP1L1
NAP1L2
NAP1L4
NASP
NAT10
NBN
NCL
NCOA1
NCOA2
NCOA3
NCOA6
NCOR1
NCOR2

NEK6
NEK9
NFRKB
NFYB
NFYC
NIPBL
NOC2L
NPAS2
NPM1
NPM2
NSD1
NSL1
OGT
PADI1
PADI2
PADI3
PADI4
PAF1
PAGR1
PAK2
PARG
PARP1
PARP2
PARP3
PAXIP1
PBK
PBRM1
PCGF1
PCGF2
PCGF3
PCGF5
PCGF6
PCNA
PDP1
PELP1
PHC1
PHC2
PHC3
PHF1
PHF10
PHF12
PHF13
PHF14
PHF19

PHF2
PHF20
PHF20L1
PHF21A
PHF8
PHIP
PIWIL4
PKM
PKN1
POGZ
POLE3
PPARGC1A
PPM1G
PPP2CA
PPP4C
PPP4R2
PRDM1
PRDM11
PRDM12
PRDM13
PRDM14
PRDM16
PRDM2
PRDM4
PRDM5
PRDM6
PRDM7
PRDM8
PRDM9
PRKAA1
PRKAA2
PRKAB1
PRKAB2
PRKAG1
PRKAG2
PRKAG3
PRKCA
PRKCB
PRKCD
PRKDC
PRMT1
PRMT2
PRMT5
PRMT6

PRMT7
PRMT8
PRMT9
PRPF31
PRR14
PSIP1
RAD51
RAD54B
RAD54L
RAD54L2
RAG1
RAG2
RAI1
RARA
RB1
RBBP4
RBBP5
RBBP7
RBX1
RCC1
RCOR1
RCOR3
REST
RING1
RLIM
RMI1
RNF168
RNF2
RNF20
RNF40
RNF8
RPS6KA3
RPS6KA4
RPS6KA5
RRP8
RSF1
RUVBL1
RUVBL2
RYBP
SAFB
SAP130
SAP18
SAP25
SAP30

SAP30L
SATB1
SATB2
SCMH1
SCML2
SCML4
SENP1
SENP3
SET
SETD1A
SETD1B
SETD2
SETD3
SETD5
SETD6
SETD7
SETD8
SETDB1
SETDB2
SETMAR
SF3B1
SF3B3
SFMBT1
SFMBT2
SFPQ
SHPRH
SIN3A
SIN3B
SIRT1
SIRT2
SIRT6
SIRT7
SKP1
SMARCA1
SMARCA2
SMARCA4
SMARCA5
SMARCAD1
SMARCAL1
SMARCB1
SMARCC1
SMARCC2
SMARCD1
SMARCD2

SMARCD3
SMARCE1
SMEK1
SMEK2
SMYD1
SMYD2
SMYD3
SMYD4
SNAI2
SP1
SP100
SP140
SPEN
SPOP
SRCAP
SRSF1
SRSF3
SS18L1
SS18L2
SSRP1
STK4
SUDS3
SUPT16H
SUPT3H
SUPT6H
SUPT7L
SUV39H1
SUV39H2
SUV420H1
SUV420H2
SUZ12
SYNCRIP
TADA1
TADA2A
TADA2B
TADA3
TAF1
TAF10
TAF12
TAF1L
TAF2
TAF3
TAF4
TAF5

TAF5L
TAF6
TAF6L
TAF7
TAF8
TAF9
TAF9B
TBL1XR1
TDG
TDRD3
TDRD7
TDRKH
TET1
TET2
TET3
TEX10
TFDP1
TFPT
TLE1
TLE2
TLE4
TLK1
TLK2
TNP1
TNP2
TONSL
TOP2A
TOP2B
TP53
TP53BP1
TRIM16
TRIM24
TRIM27
TRIM28
TRIM33
TRRAP
TSSK6
TTK
TYW5
UBE2A
UBE2B
UBE2D1
UBE2D3
UBE2E1

UBE2H
UBE2N
UBE2T
UBN1
UBR2
UBR5
UBR7
UCHL5
UHRF1
UHRF2
UIMC1
USP11
USP12
USP15
USP16
USP17L2
USP21
USP22
USP3
USP36
USP44
USP46
USP49
USP7
UTY
VDR
VPS72
VRK1
WAC
WDR5
WDR77
WDR82
WHSC1
WHSC1L1
WSB2
YAF2
YEATS2
YEATS4
YWHAB
YWHAE
YWHAZ
YY1
ZBTB16
ZBTB33

ZBTB7C
ZCWPW1
ZFP57
ZGPAT
ZHX1
ZMYM2
ZMYM3
ZMYND11
ZMYND8
ZNF217
ZNF516
ZNF532
ZNF541
ZNF592
ZNF687
ZNF711
ZNHIT1
ZRANB3
ZZZ3

table S2

ONTC	ID	Description	GeneRatio	BgRatio	pvalue	p.adjust	qvalue	geneID	Count
BP	GO:001657	histone modification	39/87	475/18614	4.22E-39	7.48E-36	6.06E-36	ARRB1/BRCC3	39
BP	GO:00182(	peptidyl-lysine modificati	23/87	374/18614	9.46E-20	8.38E-17	6.80E-17	ARRB1/BRPF3	23
BP	GO:00062	regulation of DNA repair	18/87	214/18614	5.73E-18	3.38E-15	2.74E-15	BRCC3/DPF3/	18
BP	GO:001657	histone acetylation	16/87	153/18614	1.40E-17	6.21E-15	5.03E-15	ARRB1/BRPF3	16
BP	GO:00183	internal peptidyl-lysine ac	16/87	161/18614	3.21E-17	1.14E-14	9.22E-15	ARRB1/BRPF3	16
BP	GO:00064	internal protein amino aci	16/87	163/18614	3.92E-17	1.16E-14	9.38E-15	ARRB1/BRPF3	16
BP	GO:00183	peptidyl-lysine acetylatior	16/87	172/18614	9.34E-17	2.36E-14	1.92E-14	ARRB1/BRPF3	16
BP	GO:20010	regulation of response to	19/87	311/18614	2.49E-16	5.51E-14	4.47E-14	ATM/BRCC3/	19
BP	GO:00063	chromatin remodeling	20/87	402/18614	1.92E-15	3.45E-13	2.80E-13	BAHD1/CHRA	20
BP	GO:00064	protein acetylation	16/87	208/18614	1.95E-15	3.45E-13	2.80E-13	ARRB1/BRPF3	16
BP	GO:00510	positive regulation of DN/	18/87	303/18614	2.72E-15	4.38E-13	3.55E-13	A1CF/ATM/B	18
BP	GO:00457	positive regulation of DN/	13/87	130/18614	3.37E-14	4.79E-12	3.88E-12	BRCC3/DPF3/	13
BP	GO:00435	protein acylation	16/87	250/18614	3.51E-14	4.79E-12	3.88E-12	ARRB1/BRPF3	16
BP	GO:20010	positive regulation of resp	14/87	167/18614	3.87E-14	4.90E-12	3.97E-12	ATM/BRCC3/	14
BP	GO:00439	histone H4 acetylation	8/87	63/18614	5.15E-10	6.08E-08	4.93E-08	ARRB1/EPC1/	8
BP	GO:00439	histone H3 acetylation	8/87	67/18614	8.54E-10	9.46E-08	7.67E-08	BRPF3/KAT6E	8
BP	GO:00063	double-strand break repa	13/87	303/18614	1.52E-09	1.58E-07	1.28E-07	ATM/BRCC3/	13
BP	GO:001657	histone ubiquitination	7/87	46/18614	1.76E-09	1.73E-07	1.40E-07	BRCC3/CUL4I	7
BP	GO:00103	histone monoubiquitinati	6/87	31/18614	5.87E-09	5.47E-07	4.44E-07	BRCC3/CUL4I	6
BP	GO:20007	positive regulation of dou	8/87	86/18614	6.47E-09	5.73E-07	4.65E-07	DPF3/EPC1/E	8
BP	GO:20007	regulation of double-stra	9/87	133/18614	1.18E-08	9.99E-07	8.10E-07	DPF3/EPC1/E	9
BP	GO:00064	protein methylation	10/87	182/18614	1.30E-08	1.00E-06	8.13E-07	DNMT3A/NC	10
BP	GO:00082	protein alkylation	10/87	182/18614	1.30E-08	1.00E-06	8.13E-07	DNMT3A/NC	10
BP	GO:00434	macromolecule methylati	12/87	320/18614	3.04E-08	2.24E-06	1.82E-06	DNMT3A/NC	12
BP	GO:00718	protein-DNA complex sul	11/87	263/18614	3.94E-08	2.80E-06	2.27E-06	CHRAC1/KAT	11
BP	GO:00709	demethylation	7/87	73/18614	4.84E-08	3.30E-06	2.67E-06	A1CF/APOBE	7
BP	GO:00322	methylation	12/87	365/18614	1.28E-07	8.38E-06	6.80E-06	DNMT3A/NC	12
BP	GO:00350	regulation of histone acet	6/87	53/18614	1.68E-07	1.07E-05	8.65E-06	ARRB1/NAP1	6
BP	GO:00335	histone H2A ubiquitinatio	5/87	28/18614	1.79E-07	1.10E-05	8.88E-06	BRCC3/CUL4I	5
BP	GO:20007	regulation of peptidyl-lysi	6/87	62/18614	4.36E-07	2.58E-05	2.09E-05	ARRB1/NAP1	6
BP	GO:19019	regulation of protein acet	6/87	77/18614	1.59E-06	9.06E-05	7.34E-05	ARRB1/NAP1	6
BP	GO:00355	histone H2A monoubiquit	4/87	19/18614	1.64E-06	9.06E-05	7.34E-05	BRCC3/CUL4I	4
BP	GO:00180	peptidyl-lysine methylatic	7/87	123/18614	1.75E-06	9.38E-05	7.61E-05	NCOA6/NFY(	7
BP	GO:00650	protein-DNA complex ass	9/87	242/18614	1.94E-06	0.000101	8.21E-05	CHRAC1/KAT	9
BP	GO:00065	protein monoubiquitinati	6/87	82/18614	2.31E-06	0.000115	9.34E-05	BRCC3/CUL4I	6
BP	GO:00427	mRNA transcription by RN	5/87	46/18614	2.34E-06	0.000115	9.34E-05	NCOA2/TAF4	5

BP	GO:00165	histone deubiquitination	5/87	47/18614	2.61E-06	0.000125	0.000101	BRCC3/MYSM	5
BP	GO:00165	histone methylation	7/87	133/18614	2.95E-06	0.000137	0.000111	NCOA6/NFY(	7
BP	GO:00347	nucleosome organization	7/87	138/18614	3.77E-06	0.000171	0.000139	CHRAC1/KAT	7
BP	GO:00092	mRNA transcription	5/87	51/18614	3.94E-06	0.000174	0.000141	NCOA2/TAF4	5
BP	GO:00310	regulation of histone mod	7/87	141/18614	4.34E-06	0.000186	0.000151	ARRB1/NAP1	7
BP	GO:00180	peptidyl-lysine dimethylat	4/87	24/18614	4.40E-06	0.000186	0.000151	SETD2/SETD3	4
BP	GO:00105	regulation of transpositior	4/87	28/18614	8.37E-06	0.000329	0.000267	APOBEC3B/A	4
BP	GO:00105	negative regulation of tra	4/87	28/18614	8.37E-06	0.000329	0.000267	APOBEC3B/A	4
BP	GO:20008	regulation of nucleotide-e	4/87	28/18614	8.37E-06	0.000329	0.000267	DPF3/KAT7/S	4
BP	GO:00349	histone lysine methylation	6/87	104/18614	9.21E-06	0.000355	0.000288	NCOA6/NFY(	6
BP	GO:00305	intracellular receptor sign	9/87	299/18614	1.08E-05	0.000394	0.000319	FOXA1/FOXP	9
BP	GO:00350	positive regulation of hist	4/87	30/18614	1.11E-05	0.000394	0.000319	ARRB1/NAP1	4
BP	GO:00355	monoubiquitinated histon	4/87	30/18614	1.11E-05	0.000394	0.000319	MYSM1/TAF4	4
BP	GO:00355	monoubiquitinated histon	4/87	30/18614	1.11E-05	0.000394	0.000319	MYSM1/TAF4	4
BP	GO:00321	transposition	4/87	32/18614	1.45E-05	0.000503	0.000408	APOBEC3B/A	4
BP	GO:00007	double-strand break repa	7/87	171/18614	1.54E-05	0.000524	0.000425	ATM/EPC1/E	7
BP	GO:00305	intracellular steroid horm	6/87	115/18614	1.64E-05	0.000539	0.000437	FOXA1/FOXP	6
BP	GO:00801	DNA demethylation	4/87	33/18614	1.64E-05	0.000539	0.000437	A1CF/APOBE	4
BP	GO:00007	recombinational repair	7/87	175/18614	1.79E-05	0.000576	0.000467	ATM/EPC1/E	7
BP	GO:00329	circadian regulation of ge	5/87	71/18614	2.03E-05	0.000632	0.000513	NCOA2/NPA	5
BP	GO:00063	DNA modification	6/87	120/18614	2.09E-05	0.000632	0.000513	A1CF/APOBE	6
BP	GO:00165	cytidine to uridine editing	3/87	12/18614	2.10E-05	0.000632	0.000513	A1CF/APOBE	3
BP	GO:00902	regulation of histone H4 a	3/87	12/18614	2.10E-05	0.000632	0.000513	ARRB1/SMAR	3
BP	GO:00105	regulation of double-stra	5/87	72/18614	2.17E-05	0.000641	0.00052	EPC1/EPC2/M	5
BP	GO:00355	monoubiquitinated protei	4/87	36/18614	2.34E-05	0.000664	0.000538	MYSM1/TAF4	4
BP	GO:20007	positive regulation of pep	4/87	36/18614	2.34E-05	0.000664	0.000538	ARRB1/NAP1	4
BP	GO:19037	regulation of hemopoiesis	10/87	415/18614	2.36E-05	0.000664	0.000538	BRPF3/FOXP1	10
BP	GO:00063	DNA recombination	9/87	332/18614	2.48E-05	0.000686	0.000556	ATM/EPC1/E	9
BP	GO:00355	DNA dealkylation	4/87	38/18614	2.91E-05	0.000793	0.000643	A1CF/APOBE	4
BP	GO:00450	G0 to G1 transition	4/87	41/18614	3.95E-05	0.001061	0.00086	CDK3/DPF3/S	4
BP	GO:00434	steroid hormone mediate	6/87	135/18614	4.06E-05	0.001074	0.000871	FOXA1/FOXP	6
BP	GO:00093	response to radiation	10/87	452/18614	4.86E-05	0.001267	0.001027	ATM/BRCC3/	10
BP	GO:00310	positive regulation of hist	5/87	88/18614	5.74E-05	0.001473	0.001194	ARRB1/NAP1	5
BP	GO:00033	V(D)J recombination	3/87	17/18614	6.40E-05	0.001619	0.001313	ATM/RAG1/Y	3
BP	GO:19019	positive regulation of prot	4/87	47/18614	6.81E-05	0.001676	0.001359	ARRB1/NAP1	4
BP	GO:19024	positive regulation of sten	4/87	47/18614	6.81E-05	0.001676	0.001359	SAP30/SAP3	4
BP	GO:00063	DNA-templated transcript	6/87	153/18614	8.16E-05	0.00198	0.001606	NCOA6/PPAF	6
BP	GO:00165	base conversion or substit	3/87	19/18614	9.05E-05	0.002168	0.001758	A1CF/APOBE	3

BP	GO:012016	positive regulation of colc	5/87	98/18614	9.59E-05	0.002265	0.001836	JAK2/PPARG(	5
BP	GO:001657	protein deubiquitination	6/87	159/18614	0.000101	0.002352	0.001907	BRCC3/MYSN	6
BP	GO:003969	single stranded viral RNA	3/87	20/18614	0.000106	0.002443	0.001981	APOBEC3B/A	3
BP	GO:003009	lymphocyte differentiatior	9/87	422/18614	0.000156	0.00355	0.002879	ATM/FOXP1/	9
BP	GO:004396	histone H2A acetylation	3/87	23/18614	0.000163	0.003616	0.002932	EPC1/EPC2/N	3
BP	GO:007007	histone lysine demethylati	3/87	23/18614	0.000163	0.003616	0.002932	KDM4A/KDM	3
BP	GO:003304	regulation of chromosom	7/87	250/18614	0.00017	0.003716	0.003013	ATM/DPF3/SI	7
BP	GO:000008	G1/S transition of mitotic	7/87	251/18614	0.000174	0.003762	0.00305	ATM/CDK3/C	7
BP	GO:001982	stem cell population main	6/87	177/18614	0.000181	0.003825	0.003102	KDM4C/PHF1	6
BP	GO:007064	protein modification by sr	6/87	177/18614	0.000181	0.003825	0.003102	BRCC3/MYSN	6
BP	GO:001657	histone demethylation	3/87	24/18614	0.000186	0.003876	0.003143	KDM4A/KDM	3
BP	GO:009872	maintenance of cell numb	6/87	181/18614	0.000205	0.004217	0.00342	KDM4C/PHF1	6
BP	GO:190245	negative regulation of ste	3/87	26/18614	0.000237	0.004832	0.003918	SAP30/SAP3(	3
BP	GO:004002	epigenetic regulation of g	6/87	188/18614	0.000251	0.005058	0.004102	BAHD1/DNM	6
BP	GO:000975	hormone-mediated signa	6/87	192/18614	0.000281	0.005557	0.004506	FOXA1/FOXP	6
BP	GO:190198	regulation of cell cycle ph	9/87	457/18614	0.000282	0.005557	0.004506	ATM/BRCC3/	9
BP	GO:000256	somatic diversification of i	4/87	71/18614	0.000341	0.006562	0.005321	ATM/RAG1/R	4
BP	GO:001644	somatic cell DNA recombi	4/87	71/18614	0.000341	0.006562	0.005321	ATM/RAG1/R	4
BP	GO:004484	cell cycle G1/S phase trans	7/87	281/18614	0.000346	0.006592	0.005345	ATM/CDK3/C	7
BP	GO:200003	regulation of stem cell po	4/87	72/18614	0.000359	0.006667	0.005406	SAP30/SAP3(	4
BP	GO:000648	protein demethylation	3/87	30/18614	0.000365	0.006667	0.005406	KDM4A/KDM	3
BP	GO:000821	protein dealkylation	3/87	30/18614	0.000365	0.006667	0.005406	KDM4A/KDM	3
BP	GO:003969	viral RNA genome replicat	3/87	30/18614	0.000365	0.006667	0.005406	APOBEC3B/A	3
BP	GO:190313	mononuclear cell differen	9/87	474/18614	0.000369	0.006667	0.005406	ATM/FOXP1/	9
BP	GO:000176	morphogenesis of a branc	6/87	203/18614	0.000379	0.006781	0.005499	FOXA1/KDM5	6
BP	GO:000001	regulation of DNA recomb	5/87	134/18614	0.000413	0.007321	0.005937	EPC1/EPC2/N	5
BP	GO:007138	cellular response to steroi	6/87	209/18614	0.000442	0.007759	0.006291	FOXA1/FOXP	6
BP	GO:000220	somatic diversification of i	4/87	78/18614	0.000488	0.008476	0.006873	ATM/RAG1/R	4
BP	GO:003021	T cell differentiation	7/87	300/18614	0.000512	0.008805	0.00714	FOXP1/IKZF3	7
BP	GO:007089	transcription preinitiation	4/87	83/18614	0.000618	0.010521	0.008531	SMARCB1/TA	4
BP	GO:012016	regulation of cold-induce	5/87	147/18614	0.00063	0.010637	0.008625	JAK2/PPARG(	5
BP	GO:010610	cold-induced thermogene	5/87	148/18614	0.00065	0.010865	0.00881	JAK2/PPARG(	5
BP	GO:000628	nucleotide-excision repair	4/87	85/18614	0.000676	0.011188	0.009072	DPF3/KAT7/S	4
BP	GO:007252	pyrimidine-containing co	3/87	38/18614	0.000739	0.012128	0.009834	APOBEC3B/A	3
BP	GO:007031	regulation of G0 to G1 tra	3/87	39/18614	0.000798	0.012856	0.010425	DPF3/SMARC	3
BP	GO:190516	positive regulation of dou	3/87	39/18614	0.000798	0.012856	0.010425	EPC1/EPC2/N	3
BP	GO:001802	peptidyl-lysine trimethylat	3/87	40/18614	0.00086	0.013727	0.011131	NFYC/SETD2/	3
BP	GO:003007	regulation of mitotic meta	4/87	91/18614	0.000873	0.013812	0.0112	ATM/DPF3/SI	4

BP	GO:00097	post-embryonic developn	4/87	92/18614	0.000909	0.014141	0.011466	ATM/JAK2/KD	4
BP	GO:00430	DNA methylation involvec	2/87	10/18614	0.000948	0.014141	0.011466	DNMT3A/TET	2
BP	GO:00439	histone H3-K9 acetylation	2/87	10/18614	0.000948	0.014141	0.011466	NAP1L2/SMA	2
BP	GO:00508	positive regulation of B ce	2/87	10/18614	0.000948	0.014141	0.011466	FOXP1/PRKCI	2
BP	GO:00705	regulation of vitamin D re	2/87	10/18614	0.000948	0.014141	0.011466	TRIM24/VDR	2
BP	GO:19033	regulation of fear respons	2/87	10/18614	0.000948	0.014141	0.011466	NPAS2/RAG1	2
BP	GO:19908	adaptive thermogenesis	5/87	161/18614	0.00095	0.014141	0.011466	JAK2/PPARG(	5
BP	GO:19020	regulation of metaphase/a	4/87	94/18614	0.000986	0.014554	0.011801	ATM/DPF3/SI	4
BP	GO:00070	metaphase/anaphase tran	4/87	95/18614	0.001025	0.015015	0.012175	ATM/DPF3/SI	4
BP	GO:00485	response to steroid horm	7/87	339/18614	0.001049	0.015236	0.012354	FOXA1/FOXP	7
BP	GO:00456	positive regulation of myc	3/87	43/18614	0.001063	0.015318	0.012421	DPF3/SMARC	3
BP	GO:00447	metaphase/anaphase tran	4/87	98/18614	0.001151	0.016449	0.013338	ATM/DPF3/SI	4
BP	GO:00466	response to antibiotic	3/87	47/18614	0.001378	0.018723	0.015182	JAK2/KAT7/KI	3
BP	GO:00023	immature B cell differentia	2/87	12/18614	0.001383	0.018723	0.015182	ATM/RAG1	2
BP	GO:00062	cytidine catabolic process	2/87	12/18614	0.001383	0.018723	0.015182	APOBEC3B/A	2
BP	GO:00099	cytidine deamination	2/87	12/18614	0.001383	0.018723	0.015182	APOBEC3B/A	2
BP	GO:00460	cytidine metabolic proces	2/87	12/18614	0.001383	0.018723	0.015182	APOBEC3B/A	2
BP	GO:00705	vitamin D receptor signali	2/87	12/18614	0.001383	0.018723	0.015182	TRIM24/VDR	2
BP	GO:00109	regulation of glucose met	4/87	103/18614	0.001384	0.018723	0.015182	MST1/NCOA	4
BP	GO:19019	regulation of mitotic cell c	7/87	357/18614	0.001413	0.01897	0.015382	ATM/BRCC3/	7
BP	GO:00330	regulation of sister chrom	4/87	104/18614	0.001434	0.019112	0.015497	ATM/DPF3/SI	4
BP	GO:00016	temperature homeostasis	5/87	179/18614	0.001521	0.020055	0.016261	JAK2/PPARG(	5
BP	GO:00447	mitotic cell cycle phase tr	8/87	467/18614	0.001528	0.020055	0.016261	ATM/BRCC3/	8
BP	GO:00150	DNA integration	2/87	13/18614	0.001629	0.020917	0.016961	SETMAR/SM/	2
BP	GO:00450	DNA deamination	2/87	13/18614	0.001629	0.020917	0.016961	APOBEC3B/A	2
BP	GO:00610	negative regulation of cell	2/87	13/18614	0.001629	0.020917	0.016961	FOXP1/YY1	2
BP	GO:00458	negative regulation of ge	4/87	109/18614	0.001705	0.021738	0.017626	BAHD1/DNM	4
BP	GO:00061	regulation of gluconeoge	3/87	51/18614	0.001747	0.022107	0.017926	MST1/PPARG	3
BP	GO:00454	myoblast differentiation	4/87	110/18614	0.001763	0.022159	0.017968	DPF3/HINFP/	4
BP	GO:20000	regulation of G1/S transiti	5/87	186/18614	0.0018	0.022458	0.01821	ATM/CUL4B/	5
BP	GO:00063	transcription initiation at F	4/87	111/18614	0.001823	0.022586	0.018314	PPARGC1A/T	4
BP	GO:00421	B cell activation	6/87	276/18614	0.001863	0.022923	0.018588	ATM/IKZF3/P	6
BP	GO:00461	pyrimidine ribonucleoside	2/87	14/18614	0.001895	0.022996	0.018647	APOBEC3B/A	2
BP	GO:00461	pyrimidine ribonucleoside	2/87	14/18614	0.001895	0.022996	0.018647	APOBEC3B/A	2
BP	GO:00305	intracellular estrogen rece	3/87	54/18614	0.002059	0.024826	0.02013	FOXA1/PADI	3
BP	GO:00064	protein deacetylation	4/87	116/18614	0.002141	0.025635	0.020786	HDAC11/RCC	4
BP	GO:00063	nucleosome assembly	4/87	118/18614	0.002278	0.027097	0.021972	CHRAC1/KAT	4
BP	GO:00457	negative regulation of glu	2/87	16/18614	0.002483	0.028951	0.023475	MST1/SIRT6	2

BP	GO:00458(	negative regulation of sin(	2/87	16/18614	0.002483	0.028951	0.023475	APOBEC3B/A	2
BP	GO:00461:	pyrimidine nucleoside cat:	2/87	16/18614	0.002483	0.028951	0.023475	APOBEC3B/A	2
BP	GO:00182:	peptidyl-arginine methyla	2/87	17/18614	0.002806	0.032287	0.02618	PRDM1/PRDM	2
BP	GO:00430	DNA methylation involved	2/87	17/18614	0.002806	0.032287	0.02618	DNMT3A/PIW	2
BP	GO:00076:	circadian rhythm	5/87	207/18614	0.002863	0.032728	0.026537	NCOA2/NPA:	5
BP	GO:00485:	rhythmic process	6/87	303/18614	0.002966	0.033551	0.027205	KDM5B/NCO.	6
BP	GO:00356(	protein deacylation	4/87	127/18614	0.002973	0.033551	0.027205	HDAC11/RCC	4
BP	GO:00602(	positive regulation of tran	3/87	62/18614	0.003057	0.034285	0.0278	TAF4/TAF5/T.	3
BP	GO:00063!	DNA-templated transcript	4/87	129/18614	0.003144	0.034825	0.028238	KAT7/RNF16{	4
BP	GO:00487(	embryonic skeletal system	4/87	129/18614	0.003144	0.034825	0.028238	MBTD1/PCGF	4
BP	GO:00456:	regulation of lymphocyte	5/87	212/18614	0.003171	0.034904	0.028302	IKZF3/PRDM1	5
BP	GO:19028(	regulation of cell cycle G1	5/87	214/18614	0.003301	0.035905	0.029114	ATM/CUL4B/	5
BP	GO:0051 9{	regulation of chromosom	4/87	131/18614	0.003323	0.035905	0.029114	ATM/DPF3/SI	4
BP	GO:00987:	macromolecule deacylatic	4/87	131/18614	0.003323	0.035905	0.029114	HDAC11/RCC	4
BP	GO:00515(	histone H3-K4 methylatio	3/87	64/18614	0.003345	0.035926	0.029131	NCOA6/NFY(	3
BP	GO:00315	DNA integrity checkpoint	4/87	132/18614	0.003415	0.036339	0.029466	ATM/BRCC3/	4
BP	GO:00488!	neural nucleus developme	3/87	65/18614	0.003495	0.036339	0.029466	FOXP2/PADI2	3
BP	GO:00063:	nucleosome disassembly	2/87	19/18614	0.003507	0.036339	0.029466	SMARCB1/SN	2
BP	GO:00342	negative regulation of tra	2/87	19/18614	0.003507	0.036339	0.029466	RNF168/SIRT(	2
BP	GO:00450!	regulation of single stranc	2/87	19/18614	0.003507	0.036339	0.029466	APOBEC3B/A	2
BP	GO:00453	regulation of MHC class II	2/87	19/18614	0.003507	0.036339	0.029466	JAK2/TAF7	2
BP	GO:00062	regulation of DNA replica	4/87	136/18614	0.003799	0.038822	0.03148	BRPF3/CHRA(	4
BP	GO:19021(	regulation of leukocyte di	6/87	319/18614	0.003817	0.038822	0.03148	FOXP1/IKZF3.	6
BP	GO:00083	determination of adult life	2/87	20/18614	0.003885	0.038822	0.03148	ATM/SIRT6	2
BP	GO:00181!	peptidyl-arginine modific:	2/87	20/18614	0.003885	0.038822	0.03148	PRDM1/PRDM	2
BP	GO:00424!	ribonucleoside catabolic p	2/87	20/18614	0.003885	0.038822	0.03148	APOBEC3B/A	2
BP	GO:00453	MHC class II biosynthetic	2/87	20/18614	0.003885	0.038822	0.03148	JAK2/TAF7	2
BP	GO:00074!	mesoderm development	4/87	137/18614	0.0039	0.038822	0.03148	EYA2/IKZF3/J	4
BP	GO:00511:	RNA polymerase II preiniti	3/87	68/18614	0.00397	0.038869	0.031517	TAF4/TAF5/T.	3
BP	GO:00611{	mammary gland epitheliu	3/87	68/18614	0.00397	0.038869	0.031517	JAK2/KDM5B.	3
BP	GO:20001	positive regulation of DN/	3/87	68/18614	0.00397	0.038869	0.031517	TAF4/TAF5/T.	3
BP	GO:00108:	negative regulation of glu	2/87	21/18614	0.004281	0.040387	0.032748	PRKCB/SIRT6	2
BP	GO:00217!	striatum development	2/87	21/18614	0.004281	0.040387	0.032748	FOXP1/FOXP.	2
BP	GO:00329{	protein-DNA complex dis	2/87	21/18614	0.004281	0.040387	0.032748	SMARCB1/SN	2
BP	GO:00610!	regulation of cell growth i	2/87	21/18614	0.004281	0.040387	0.032748	FOXP1/YY1	2
BP	GO:00063(	DNA alkylation	3/87	70/18614	0.004308	0.040387	0.032748	DNMT3A/PIW	3
BP	GO:00063(	DNA methylation	3/87	70/18614	0.004308	0.040387	0.032748	DNMT3A/PIW	3
BP	GO:00322(	positive regulation of telo	3/87	70/18614	0.004308	0.040387	0.032748	ATM/SIRT6/Y	3

BP	GO:00436{	post-translational protein	3/87	70/18614	0.004308	0.040387	0.032748	JAK2/PRKCB/	3
BP	GO:00102:	response to ionizing radia	4/87	143/18614	0.004541	0.04235	0.03434	ATM/BRCC3/	4
BP	GO:00487:	gland development	7/87	441/18614	0.004584	0.042441	0.034413	ATM/FOXA1/	7
BP	GO:00331²	regulation of intracellular	3/87	72/18614	0.004662	0.042441	0.034413	FOXA1/FOXP	3
BP	GO:00459:	positive regulation of DN/	3/87	72/18614	0.004662	0.042441	0.034413	EPC1/EPC2/M	3
BP	GO:00602(	regulation of transcription	3/87	72/18614	0.004662	0.042441	0.034413	TAF4/TAF5/T.	3
BP	GO:00327{	negative regulation of DN	2/87	22/18614	0.004694	0.042441	0.034413	RNF168/SIRT(	2
BP	GO:00604²	branching involved in mai	2/87	22/18614	0.004694	0.042441	0.034413	KDM5B/VDR	2
BP	GO:00605(	epithelial tube morphoge	6/87	334/18614	0.004768	0.042892	0.03478	FOXA1/FOXP	6
BP	GO:00456(	regulation of myoblast dif	3/87	74/18614	0.005033	0.045046	0.036526	DPF3/SMARC	3
BP	GO:00508!	regulation of B cell recept	2/87	23/18614	0.005126	0.045415	0.036825	FOXP1/PRKC	2
BP	GO:00713(	cellular response to vitam	2/87	23/18614	0.005126	0.045415	0.036825	TRIM24/VDR	2
BP	GO:00062:	pyrimidine nucleoside me	2/87	24/18614	0.005575	0.049148	0.039852	APOBEC3B/A	2
BP	GO:00106;	regulation of cellular carb	4/87	152/18614	0.00563	0.04939	0.040049	MST1/NCOA:	4
BP	GO:00301{	B cell differentiation	4/87	153/18614	0.005761	0.050289	0.040778	ATM/IKZF3/R	4
BP	GO:00094:	response to UV	4/87	154/18614	0.005894	0.051197	0.041514	CUL4B/SIRT6/	4
BP	GO:00488(	stem cell differentiation	5/87	247/18614	0.006039	0.051717	0.041935	FOXA1/KDM²	5
BP	GO:00017;	natural killer cell differenti	2/87	25/18614	0.006041	0.051717	0.041935	KAT7/PRDM1	2
BP	GO:00091(	nucleoside catabolic proc	2/87	25/18614	0.006041	0.051717	0.041935	APOBEC3B/A	2
BP	GO:00518(	protein autoubiquitinatior	3/87	80/18614	0.006253	0.053014	0.042987	BRCC3/RAG1	3
BP	GO:20001²	regulation of DNA-templa	3/87	80/18614	0.006253	0.053014	0.042987	TAF4/TAF5/T.	3
BP	GO:00487!	branching morphogenesis	4/87	157/18614	0.006305	0.053202	0.043139	FOXA1/KDM!	4
BP	GO:00508!	positive regulation of anti	2/87	26/18614	0.006525	0.054799	0.044434	FOXP1/PRKC	2
BP	GO:00320:	positive regulation of insu	3/87	83/18614	0.006923	0.057593	0.0467	JAK2/PRKCB/	3
BP	GO:00725:	pyrimidine-containing coi	3/87	83/18614	0.006923	0.057593	0.0467	APOBEC3B/A	3
BP	GO:00316(	response to nutrient levels	7/87	477/18614	0.006968	0.057638	0.046736	DNMT3A/KD	7
BP	GO:00215²	subpallium development	2/87	27/18614	0.007026	0.057638	0.046736	FOXP1/FOXP.	2
BP	GO:00458:	transcription initiation-co	2/87	27/18614	0.007026	0.057638	0.046736	KAT7/PADI2	2
BP	GO:00060!	gluconeogenesis	3/87	84/18614	0.007155	0.05843	0.047378	MST1/PPARG	3
BP	GO:00447;	mitotic DNA integrity che	3/87	85/18614	0.007392	0.060089	0.048723	ATM/BRCC3/	3
BP	GO:00321(	regulation of response to	2/87	28/18614	0.007543	0.060484	0.049044	TRIM24/VDR	2
BP	GO:00321(	regulation of response to	2/87	28/18614	0.007543	0.060484	0.049044	TRIM24/VDR	2
BP	GO:00607(	regulation of androgen re	2/87	28/18614	0.007543	0.060484	0.049044	FOXP1/KDM²	2
BP	GO:00193:	hexose biosynthetic proce	3/87	87/18614	0.00788	0.062901	0.051004	MST1/PPARG	3
BP	GO:00616²	histone H3-K9 modificatic	2/87	29/18614	0.008078	0.064188	0.052047	NAP1L2/SMA	2
BP	GO:00332;	response to vitamin	3/87	88/18614	0.008131	0.064325	0.052158	DNMT3A/TRI	3
BP	GO:00346!	nucleobase-containing sn	2/87	30/18614	0.008629	0.067061	0.054377	APOBEC3B/A	2
BP	GO:00550:	negative regulation of car	2/87	30/18614	0.008629	0.067061	0.054377	FOXP1/YY1	2

BP	GO:00606(	mammary gland duct mo	2/87	30/18614	0.008629	0.067061	0.054377	KDM5B/VDR	2
BP	GO:00611	negative regulation of hea	2/87	30/18614	0.008629	0.067061	0.054377	FOXP1/YY1	2
BP	GO:00510	positive regulation of DN/	5/87	271/18614	0.008836	0.068373	0.055441	FOXA1/JAK2/	5
BP	GO:00456	positive regulation of neu	3/87	92/18614	0.009182	0.069936	0.056709	FOXA1/KDM4	3
BP	GO:00032	physiological muscle hype	2/87	31/18614	0.009196	0.069936	0.056709	FOXP1/YY1	2
BP	GO:00033(	physiological cardiac mus	2/87	31/18614	0.009196	0.069936	0.056709	FOXP1/YY1	2
BP	GO:00610	cell growth involved in ca	2/87	31/18614	0.009196	0.069936	0.056709	FOXP1/YY1	2
BP	GO:00094	RNA modification	4/87	177/18614	0.009537	0.072222	0.058562	A1CF/APOBE	4
BP	GO:00165	histone deacetylation	3/87	94/18614	0.009736	0.072635	0.058897	HDAC11/RCC	3
BP	GO:00101	response to X-ray	2/87	32/18614	0.009779	0.072635	0.058897	ATM/BRCC3	2
BP	GO:00310	negative regulation of his	2/87	32/18614	0.009779	0.072635	0.058897	SMARCB1/TA	2
BP	GO:00712	cellular response to vitam	2/87	32/18614	0.009779	0.072635	0.058897	TRIM24/VDR	2
BP	GO:00062	DNA replication	5/87	278/18614	0.009797	0.072635	0.058897	BRPF3/CHRA	5
BP	GO:00487(	embryonic skeletal system	3/87	95/18614	0.01002	0.07398	0.059987	PCGF2/SATB2	3
BP	GO:00455	regulation of T cell differe	4/87	180/18614	0.0101	0.074259	0.060214	PRDM1/RAG1	4
BP	GO:00315	heterochromatin formatio	3/87	96/18614	0.010309	0.075173	0.060955	BAHD1/DNM	3
BP	GO:00463	monosaccharide biosynth	3/87	96/18614	0.010309	0.075173	0.060955	MST1/PPARG	3
BP	GO:00063(	DNA catabolic process	2/87	33/18614	0.010379	0.075375	0.061119	ATM/SETMAI	2
BP	GO:00061(	regulation of carbohydrat	4/87	183/18614	0.010683	0.077268	0.062653	MST1/NCOA	4
BP	GO:00017(	in utero embryonic develo	6/87	398/18614	0.010868	0.078285	0.063478	HINFP/KDM4	6
BP	GO:19016	glycosyl compound catab	2/87	34/18614	0.010994	0.078875	0.063957	APOBEC3B/A	2
BP	GO:00033(	cardiac muscle hypertropl	3/87	99/18614	0.011204	0.080055	0.064913	FOXP1/KDM4	3
BP	GO:00060(	glucose metabolic proces	4/87	187/18614	0.011495	0.08101	0.065688	MST1/NCOA	4
BP	GO:00301(	natural killer cell activatio	3/87	100/18614	0.011512	0.08101	0.065688	JAK2/KAT7/P	3
BP	GO:00305	negative regulation of tra	3/87	100/18614	0.011512	0.08101	0.065688	SAP30/SAP3	3
BP	GO:00018	maternal placenta develo	2/87	35/18614	0.011626	0.08101	0.065688	PRDM1/VDR	2
BP	GO:00070	mitotic G2 DNA damage	2/87	35/18614	0.011626	0.08101	0.065688	ATM/BRCC3	2
BP	GO:00091	ribonucleoside metabolic	2/87	35/18614	0.011626	0.08101	0.065688	APOBEC3B/A	2
BP	GO:00000	cell cycle checkpoint sign	4/87	188/18614	0.011703	0.08101	0.065688	ATM/BRCC3/	4
BP	GO:00611	morphogenesis of a branc	4/87	188/18614	0.011703	0.08101	0.065688	FOXA1/KDM5	4
BP	GO:00103	regulation of G2/M transit	3/87	101/18614	0.011825	0.081219	0.065857	ATM/BRCC3/	3
BP	GO:00432	regulation of carbohydrat	3/87	101/18614	0.011825	0.081219	0.065857	MST1/PPARG	3
BP	GO:00148	striated muscle hypertrop	3/87	102/18614	0.012143	0.08308	0.067366	FOXP1/KDM4	3
BP	GO:00106	negative regulation of car	2/87	36/18614	0.012272	0.083642	0.067822	FOXP1/YY1	2
BP	GO:00714	cellular response to radiat	4/87	191/18614	0.012345	0.083812	0.06796	ATM/BRCC3/	4
BP	GO:00148	muscle hypertrophy	3/87	104/18614	0.012794	0.086167	0.069869	FOXP1/KDM4	3
BP	GO:00322(	regulation of telomere ma	3/87	104/18614	0.012794	0.086167	0.069869	ATM/SIRT6/Y	3
BP	GO:00332	response to vitamin D	2/87	37/18614	0.012935	0.086167	0.069869	TRIM24/VDR	2

BP	GO:00457	negative regulation of No	2/87	37/18614	0.012935	0.086167	0.069869	ARRB1/CDK3	2
BP	GO:00703	lens fiber cell differentiatio	2/87	37/18614	0.012935	0.086167	0.069869	KDM5B/TDRD	2
BP	GO:00456	regulation of neuron diffe	4/87	194/18614	0.013008	0.086331	0.070003	FOXA1/HMG	4
BP	GO:00327	regulation of DNA-templa	3/87	105/18614	0.013126	0.086467	0.070112	KAT7/RNF16	3
BP	GO:00708	heterochromatin organiza	3/87	105/18614	0.013126	0.086467	0.070112	BAHD1/DNM	3
BP	GO:00016	behavioral fear response	2/87	38/18614	0.013612	0.088561	0.071811	NPAS2/RAG1	2
BP	GO:00147	negative regulation of mu	2/87	38/18614	0.013612	0.088561	0.071811	FOXP1/YY1	2
BP	GO:00702	negative regulation of lym	2/87	38/18614	0.013612	0.088561	0.071811	FOXP1/RAG1	2
BP	GO:00025	immune system developm	4/87	197/18614	0.013694	0.088561	0.071811	ATM/RAG1/R	4
BP	GO:00064	RNA localization	4/87	197/18614	0.013694	0.088561	0.071811	A1CF/ATM/S	4
BP	GO:00468	regulation of nucleocytop	3/87	107/18614	0.013806	0.088639	0.071874	JAK2/SETD2/	3
BP	GO:00902	positive regulation of pep	3/87	107/18614	0.013806	0.088639	0.071874	JAK2/PRKCB/	3
BP	GO:00022	behavioral defense respor	2/87	39/18614	0.014305	0.09053	0.073407	NPAS2/RAG1	2
BP	GO:00457	positive regulation of DN	2/87	39/18614	0.014305	0.09053	0.073407	BRPF3/KAT7	2
BP	GO:00466	negative regulation of org	2/87	39/18614	0.014305	0.09053	0.073407	FOXP1/YY1	2
BP	GO:00989	postsynaptic signal transd	2/87	39/18614	0.014305	0.09053	0.073407	JAK2/PRKCB	2
BP	GO:00027	positive regulation of pep	3/87	109/18614	0.014506	0.09115	0.07391	JAK2/PRKCB/	3
BP	GO:00421	macrophage activation	3/87	109/18614	0.014506	0.09115	0.07391	ATM/FOXP1/	3
BP	GO:00605	muscle tissue developmer	6/87	426/18614	0.014784	0.092573	0.075063	EYA2/FOXP1/	6
BP	GO:00063	transcription elongation b	3/87	110/18614	0.014863	0.092737	0.075197	RNF168/SETD	3
BP	GO:20012	positive regulation of chro	3/87	111/18614	0.015225	0.094664	0.076759	ATM/SIRT6/Y	3
BP	GO:19027	regulation of cell cycle G2	3/87	112/18614	0.015592	0.096608	0.078335	ATM/BRCC3/	3
BP	GO:00511	negative regulation of stri	2/87	41/18614	0.015735	0.097151	0.078776	FOXP1/YY1	2
BP	GO:00070	chromosome segregation	6/87	435/18614	0.016229	0.099853	0.080966	ATM/DPF3/N	6
BP	GO:00455	positive regulation of T ce	3/87	116/18614	0.017111	0.104523	0.084753	RAG1/SMAR	3
BP	GO:00486	negative regulation of dev	3/87	116/18614	0.017111	0.104523	0.084753	FOXP1/RAI1/	3
BP	GO:00316	cellular response to nutrie	2/87	43/18614	0.017224	0.104523	0.084753	TRIM24/VDR	2
BP	GO:00328	regulation of natural killer	2/87	43/18614	0.017224	0.104523	0.084753	JAK2/PRDM1	2
BP	GO:00224	positive regulation of cell-	5/87	321/18614	0.017311	0.104691	0.084889	FOXA1/JAK2/	5
BP	GO:00094	response to light stimulus	5/87	323/18614	0.017732	0.106876	0.086662	CUL4B/RAG1	5
BP	GO:00517	biological process involve	3/87	118/18614	0.0179	0.107333	0.087032	JAK2/SAP30/	3
BP	GO:00217	substantia nigra developm	2/87	44/18614	0.01799	0.107333	0.087032	PADI2/SUDS	2
BP	GO:00425	fear response	2/87	44/18614	0.01799	0.107333	0.087032	NPAS2/RAG1	2
BP	GO:00106	negative regulation of cell	2/87	45/18614	0.01877	0.11124	0.0902	MST1/SIRT6	2
BP	GO:00604	mammary gland morphog	2/87	45/18614	0.01877	0.11124	0.0902	KDM5B/VDR	2
BP	GO:00435	muscle adaptation	3/87	121/18614	0.019122	0.112948	0.091585	FOXP1/KDM	3
BP	GO:00000	DNA damage checkpoint	3/87	123/18614	0.019962	0.117126	0.094972	ATM/BRCC3/	3
BP	GO:00226	gland morphogenesis	3/87	123/18614	0.019962	0.117126	0.094972	FOXA1/KDM	3

BP	GO:00017	microglial cell activation	2/87	47/18614	0.020373	0.118751	0.096291	ATM/JAK2	2
BP	GO:00305	androgen receptor signali	2/87	47/18614	0.020373	0.118751	0.096291	FOXP1/KDM4	2
BP	GO:00485	thymus development	2/87	48/18614	0.021195	0.122736	0.099521	ATM/RAG1	2
BP	GO:00609	coronary vasculature deve	2/87	48/18614	0.021195	0.122736	0.099521	PRDM1/SETD	2
BP	GO:00485	camera-type eye morpho	3/87	127/18614	0.021701	0.124709	0.101121	PRDM1/TDRD	3
BP	GO:00550	cardiac muscle cell differe	3/87	127/18614	0.021701	0.124709	0.101121	FOXP1/SIRT6	3
BP	GO:00510	regulation of DNA-bindin	6/87	465/18614	0.021747	0.124709	0.101121	ARRB1/FOXA	6
BP	GO:00193	hexose metabolic process	4/87	228/18614	0.022147	0.126593	0.102649	MST1/NCOA	4
BP	GO:00190	viral genome replication	3/87	129/18614	0.022601	0.128774	0.104418	APOBEC3B/A	3
BP	GO:00316	cellular response to nutrie	4/87	230/18614	0.02278	0.12938	0.104909	PRKAB1/PRK	4
BP	GO:00022	leukocyte activation invol	2/87	50/18614	0.02288	0.12953	0.10503	ATM/JAK2	2
BP	GO:00456	positive regulation of lym	3/87	130/18614	0.023058	0.130126	0.105514	RAG1/SMARC	3
BP	GO:00448	mitotic G2/M transition ch	2/87	51/18614	0.023742	0.132717	0.107615	ATM/BRCC3	2
BP	GO:00459	negative regulation of car	2/87	51/18614	0.023742	0.132717	0.107615	MST1/SIRT6	2
BP	GO:00606	adipose tissue developme	2/87	51/18614	0.023742	0.132717	0.107615	NCOA2/PPAR	2
BP	GO:00621	cellular response to chem	5/87	349/18614	0.023846	0.132877	0.107744	ATM/FOXP1/	5
BP	GO:19001	regulation of protein local	3/87	134/18614	0.024939	0.138532	0.11233	JAK2/KAT7/SI	3
BP	GO:00457	positive regulation of cell	6/87	482/18614	0.02538	0.140544	0.113961	ATM/FOXA1/	6
BP	GO:20006	positive regulation of sten	2/87	53/18614	0.025507	0.140805	0.114173	KAT7/SIRT6	2
BP	GO:00000	G2/M transition of mitotic	3/87	136/18614	0.025909	0.14214	0.115255	ATM/BRCC3/	3
BP	GO:00308	mammary gland developr	3/87	136/18614	0.025909	0.14214	0.115255	JAK2/KDM5B	3
BP	GO:00442	cellular nitrogen compour	6/87	486/18614	0.026291	0.143789	0.116593	A1CF/APOBE	6
BP	GO:00711	protein localization to chr	2/87	54/18614	0.026409	0.143989	0.116754	SETD2/SIRT6	2
BP	GO:00467	heterocycle catabolic proc	6/87	488/18614	0.026754	0.145426	0.11792	A1CF/APOBE	6
BP	GO:00468	positive regulation of horr	3/87	138/18614	0.0269	0.145769	0.118198	JAK2/PRKCB/	3
BP	GO:00091	nucleoside metabolic proc	2/87	55/18614	0.027323	0.147164	0.119329	APOBEC3B/A	2
BP	GO:00619	glial cell activation	2/87	55/18614	0.027323	0.147164	0.119329	ATM/JAK2	2
BP	GO:00070	mitotic cell cycle checkpoi	3/87	140/18614	0.02791	0.14987	0.121524	ATM/BRCC3/	3
BP	GO:00450	negative regulation of vira	2/87	56/18614	0.028251	0.150784	0.122264	APOBEC3B/A	2
BP	GO:20001	negative regulation of leu	2/87	56/18614	0.028251	0.150784	0.122264	FOXP1/RAG1	2
BP	GO:00468	regulation of hormone se	4/87	247/18614	0.028615	0.152268	0.123468	JAK2/KDM5B	4
BP	GO:00425	glucose homeostasis	4/87	248/18614	0.028983	0.153767	0.124683	FOXA1/PPAR	4
BP	GO:00015	ovarian follicle developme	2/87	57/18614	0.029191	0.153893	0.124786	ATM/TAF4	2
BP	GO:00310	regulation of histone meth	2/87	57/18614	0.029191	0.153893	0.124786	PHF19/SMAR	2
BP	GO:00335	carbohydrate homeostasis	4/87	249/18614	0.029354	0.153893	0.124786	FOXA1/PPAR	4
BP	GO:00508	positive regulation of T ce	4/87	249/18614	0.029354	0.153893	0.124786	JAK2/RAG1/S	4
BP	GO:00008	sister chromatid segregati	4/87	250/18614	0.029728	0.155395	0.126003	ATM/DPF3/SI	4
BP	GO:00423	regulation of protein impo	2/87	58/18614	0.030143	0.157097	0.127384	JAK2/SIRT6	2

BP	GO:00507	positive regulation of prot	3/87	146/18614	0.031062	0.161176	0.130691	JAK2/PRKCB/	3
BP	GO:00702	regulation of lymphocyte	2/87	59/18614	0.031107	0.161176	0.130691	FOXP1/RAG1	2
BP	GO:00508	regulation of T cell activat	5/87	377/18614	0.031802	0.164293	0.133218	JAK2/PRDM1	5
BP	GO:00059	monosaccharide metaboli	4/87	256/18614	0.032032	0.164669	0.133523	MST1/NCOA	4
BP	GO:00468	positive regulation of nucl	2/87	60/18614	0.032084	0.164669	0.133523	JAK2/SIRT6	2
BP	GO:00170	regulation of transforming	3/87	148/18614	0.032153	0.164669	0.133523	SAP30/SAP30	3
BP	GO:00601	maternal process involved	2/87	61/18614	0.033073	0.168889	0.136945	PRDM1/VDR	2
BP	GO:00147	striated muscle tissue dev	4/87	259/18614	0.033223	0.16917	0.137173	EYA2/FOXP1/	4
BP	GO:00448	cell cycle G2/M phase trar	3/87	151/18614	0.033827	0.171259	0.138867	ATM/BRCC3/	3
BP	GO:19038	regulation of cellular resp	3/87	151/18614	0.033827	0.171259	0.138867	SAP30/SAP30	3
BP	GO:00316	cellular response to extrac	4/87	261/18614	0.034031	0.171527	0.139084	PRKAB1/PRK	4
BP	GO:00164	somatic recombination of	2/87	62/18614	0.034073	0.171527	0.139084	RNF168/YY1	2
BP	GO:00018	placenta development	3/87	152/18614	0.034394	0.172653	0.139997	PRDM1/SETD	3
BP	GO:00513	positive regulation of oxid	2/87	63/18614	0.035085	0.175625	0.142407	PDP1/VDR	2
BP	GO:00063	double-strand break repa	2/87	64/18614	0.036109	0.180241	0.14615	ATM/SETMAI	2
BP	GO:00007	telomere maintenance	3/87	156/18614	0.036715	0.182236	0.147767	ATM/SIRT6/Y	3
BP	GO:00075	response to nutrient	3/87	156/18614	0.036715	0.182236	0.147767	DNMT3A/TRI	3
BP	GO:00109	negative regulation of G2	2/87	65/18614	0.037145	0.182327	0.147842	ATM/BRCC3	2
BP	GO:00308	regulation of B cell prolife	2/87	65/18614	0.037145	0.182327	0.147842	ATM/IKZF3	2
BP	GO:00508	regulation of antigen rece	2/87	65/18614	0.037145	0.182327	0.147842	FOXP1/PRKC	2
BP	GO:19053	regulation of morphogen	2/87	65/18614	0.037145	0.182327	0.147842	FOXP1/SIRT6	2
BP	GO:00485	eye morphogenesis	3/87	157/18614	0.037307	0.182618	0.148078	PRDM1/TDRI	3
BP	GO:19030	positive regulation of leuk	4/87	273/18614	0.039119	0.19096	0.154842	JAK2/RAG1/S	4
BP	GO:19027	negative regulation of cell	2/87	67/18614	0.039249	0.191071	0.154931	ATM/BRCC3	2
BP	GO:19019	negative regulation of cell	4/87	274/18614	0.039561	0.191539	0.155311	ATM/BRCC3/	4
BP	GO:00350	cardiocyte differentiation	3/87	161/18614	0.039726	0.191539	0.155311	FOXP1/SIRT6	3
BP	GO:00511	regulation of muscle cell c	3/87	161/18614	0.039726	0.191539	0.155311	FOXP1/SETD3	3
BP	GO:00614	connective tissue develop	4/87	275/18614	0.040007	0.191539	0.155311	FOXA1/NCO	4
BP	GO:00164	somatic diversification of i	2/87	68/18614	0.040318	0.191539	0.155311	RNF168/YY1	2
BP	GO:00169	protein sumoylation	2/87	68/18614	0.040318	0.191539	0.155311	CBX4/HMG2	2
BP	GO:00550	regulation of cardiac mus	2/87	68/18614	0.040318	0.191539	0.155311	FOXP1/YY1	2
BP	GO:00709	protein K48-linked ubiqui	2/87	68/18614	0.040318	0.191539	0.155311	UBE2A/UBE2	2
BP	GO:19028	regulation of miRNA trans	2/87	68/18614	0.040318	0.191539	0.155311	FOXA1/YY1	2
BP	GO:00507	regulation of insulin secre	3/87	163/18614	0.040964	0.194087	0.157377	JAK2/PRKCB/	3
BP	GO:00106	regulation of cardiac mus	2/87	69/18614	0.041398	0.195101	0.158199	FOXP1/YY1	2
BP	GO:00616	miRNA transcription	2/87	69/18614	0.041398	0.195101	0.158199	FOXA1/YY1	2
BP	GO:00511	negative regulation of mu	2/87	70/18614	0.042489	0.199711	0.161937	FOXP1/YY1	2
BP	GO:00451	cell fate commitment	4/87	281/18614	0.042741	0.200361	0.162464	EYA2/FOXA1/	4

BP	GO:00456(	positive regulation of fat c	2/87	71/18614	0.043591	0.203518	0.165024	SIRT6/ZBTB7(	2
BP	GO:00147	regulation of muscle hype	2/87	72/18614	0.044703	0.203518	0.165024	FOXP1/YY1	2
BP	GO:00901(	negative regulation of tra	3/87	169/18614	0.044796	0.203518	0.165024	SAP30/SAP3(	3
BP	GO:00018(	NK T cell differentiation	1/87	10/18614	0.045779	0.203518	0.165024	PRDM1	1
BP	GO:00082(	intracellular mRNA localiz	1/87	10/18614	0.045779	0.203518	0.165024	A1CF	1
BP	GO:00105	positive regulation of plat	1/87	10/18614	0.045779	0.203518	0.165024	JAK2	1
BP	GO:00302	autophagy of peroxisome	1/87	10/18614	0.045779	0.203518	0.165024	ATM	1
BP	GO:00328	positive regulation of natu	1/87	10/18614	0.045779	0.203518	0.165024	JAK2	1
BP	GO:00335	histone H2B ubiquitinatio	1/87	10/18614	0.045779	0.203518	0.165024	UBE2E1	1
BP	GO:00361	histone H3-K9 trimethylat	1/87	10/18614	0.045779	0.203518	0.165024	SMARCB1	1
BP	GO:00510	nuclear membrane disass	1/87	10/18614	0.045779	0.203518	0.165024	PRKCB	1
BP	GO:00514(	response to nitrosative str	1/87	10/18614	0.045779	0.203518	0.165024	ATM	1
BP	GO:00605	prostate glandular acinus	1/87	10/18614	0.045779	0.203518	0.165024	FOXA1	1
BP	GO:00700	regulation of chemokine-	1/87	10/18614	0.045779	0.203518	0.165024	PADI2	1
BP	GO:00704	regulation of uterine smo	1/87	10/18614	0.045779	0.203518	0.165024	SETD3	1
BP	GO:00902	chromosome localization	1/87	10/18614	0.045779	0.203518	0.165024	ATM	1
BP	GO:19018	positive regulation of tran	1/87	10/18614	0.045779	0.203518	0.165024	SMARCB1	1
BP	GO:19028	positive regulation of resp	1/87	10/18614	0.045779	0.203518	0.165024	FOXP1	1
BP	GO:20012(	positive regulation of cyst	1/87	10/18614	0.045779	0.203518	0.165024	JAK2	1
BP	GO:00507	regulation of behavior	2/87	73/18614	0.045826	0.203518	0.165024	NPAS2/RAG1	2
BP	GO:19000	regulation of cellular resp	2/87	73/18614	0.045826	0.203518	0.165024	NCOA2/PRK(	2
BP	GO:00604	regulation of heart growth	2/87	74/18614	0.046959	0.206815	0.167697	FOXP1/YY1	2
BP	GO:19035	regulation of ATP metabo	2/87	74/18614	0.046959	0.206815	0.167697	PPARGC1A/S	2
BP	GO:00345	cellular response to oxida	4/87	290/18614	0.047035	0.206815	0.167697	FOXP1/JAK2/	4
BP	GO:00442(	cellular carbohydrate met	4/87	290/18614	0.047035	0.206815	0.167697	MST1/NCOA	4
BP	GO:00714	cellular response to ionizi	2/87	75/18614	0.048102	0.210715	0.17086	ATM/BRCC3	2
BP	GO:00466	alpha-beta T cell activatio	3/87	175/18614	0.048801	0.210715	0.17086	FOXP1/JAK2/	3
BP	GO:01500	neuroinflammatory respo	2/87	76/18614	0.049256	0.210715	0.17086	ATM/JAK2	2
BP	GO:00486(	regulation of smooth mus	3/87	176/18614	0.049485	0.210715	0.17086	FOXP1/JAK2/	3
CC	GO:00001	histone acetyltransferase	9/86	93/19518	2.90E-10	3.45E-08	2.01E-08	BRPF3/EPC1/	9
CC	GO:00312	protein acetyltransferase	9/86	103/19518	7.30E-10	3.45E-08	2.01E-08	BRPF3/EPC1/	9
CC	GO:19024	acetyltransferase complex	9/86	104/19518	7.97E-10	3.45E-08	2.01E-08	BRPF3/EPC1/	9
CC	GO:00001	histone deacetylase comp	7/86	79/19518	5.64E-08	1.83E-06	1.07E-06	HDAC11/RC(	7
CC	GO:00007	heterochromatin	6/86	81/19518	1.53E-06	3.97E-05	2.32E-05	CHRAC1/DN	6
CC	GO:00057	pericentric heterochromat	4/86	30/19518	8.82E-06	0.000191	0.000111	CHRAC1/KD	4
CC	GO:00007	chromosome, centromeri	8/86	249/19518	1.41E-05	0.000262	0.000153	CHRAC1/DN	8
CC	GO:00350(	brahma complex	3/86	14/19518	2.90E-05	0.000472	0.000275	DPF3/SMAR(	3
CC	GO:00986	chromosomal region	9/86	390/19518	5.48E-05	0.000683	0.000398	ATM/CHRAC	9

CC	GO:00706(	SWI/SNF superfamily-type	5/86	93/19518	5.66E-05	0.000683	0.000398	CHRAC1/DPF	5
CC	GO:000022	nuclear chromosome	7/86	223/19518	5.78E-05	0.000683	0.000398	CHRAC1/SAP	7
CC	GO:003327	transcription factor TFTC (	3/86	18/19518	6.42E-05	0.000696	0.000406	TAF4/TAF5/T.	3
CC	GO:001658	Sin3 complex	3/86	22/19518	0.00012	0.001085	0.000633	SAP30/SAP3(	3
CC	GO:007082	Sin3-type complex	3/86	22/19518	0.00012	0.001085	0.000633	SAP30/SAP3(	3
CC	GO:009057	RNA polymerase II transcr	7/86	254/19518	0.00013	0.001085	0.000633	NCOA2/NFY(	7
CC	GO:00007S	euchromatin	4/86	60/19518	0.000142	0.001085	0.000633	DNMT3A/JAK	4
CC	GO:00350S	histone methyltransferase	4/86	60/19518	0.000142	0.001085	0.000633	NCOA6/PRDI	4
CC	GO:004307	germ cell nucleus	4/86	67/19518	0.000218	0.001574	0.000918	SMARCB1/TA	4
CC	GO:00163(	nuclear matrix	5/86	127/19518	0.000246	0.001685	0.000982	DNMT3A/SA	5
CC	GO:001651	SWI/SNF complex	3/86	30/19518	0.000308	0.001818	0.00106	DPF3/SMAR(	3
CC	GO:00352(	NuA4 histone acetyltransf	3/86	30/19518	0.000308	0.001818	0.00106	EPC1/EPC2/M	3
CC	GO:00431{	H4/H2A histone acetyltrar	3/86	30/19518	0.000308	0.001818	0.00106	EPC1/EPC2/M	3
CC	GO:00008(	sex chromosome	3/86	31/19518	0.00034	0.001919	0.001119	DNMT3A/PC(	3
CC	GO:00616S	transferase complex, trans	7/86	306/19518	0.000405	0.00215	0.001253	CDK3/CHRA(	7
CC	GO:00001!	ubiquitin ligase complex	7/86	307/19518	0.000413	0.00215	0.001253	BRCC3/CBX4/	7
CC	GO:00016!	fibrillar center	5/86	146/19518	0.000468	0.002165	0.001262	FOXA1/KDM4	5
CC	GO:00329S	protein-DNA complex	6/86	225/19518	0.000479	0.002165	0.001262	EPC1/EPC2/K	6
CC	GO:00343S	nuclear periphery	5/86	147/19518	0.000483	0.002165	0.001262	DNMT3A/SA	5
CC	GO:19049	ATPase complex	5/86	147/19518	0.000483	0.002165	0.001262	CHRAC1/DPF	5
CC	GO:003151	PcG protein complex	3/86	37/19518	0.000576	0.002496	0.001455	CBX4/PCGF2/	3
CC	GO:00704(	SAGA-type complex	3/86	39/19518	0.000673	0.002823	0.001646	TAF4/TAF5/T.	3
CC	GO:00347(	methyltransferase comple	4/86	92/19518	0.000731	0.002969	0.001731	NCOA6/PRDI	4
CC	GO:01400S	bBAF complex	2/86	10/19518	0.000844	0.003325	0.001938	SMARCB1/SM	2
CC	GO:00001!	nuclear ubiquitin ligase co	3/86	44/19518	0.00096	0.003566	0.002079	BRCC3/CBX4/	3
CC	GO:19025(	H4 histone acetyltransfera	3/86	44/19518	0.00096	0.003566	0.002079	EPC1/EPC2/M	3
CC	GO:00056(	transcription factor TFIID (	3/86	45/19518	0.001025	0.003703	0.002159	TAF4/TAF5/T.	3
CC	GO:00907	site of DNA damage	4/86	110/19518	0.001422	0.004962	0.002893	KAT7/RNF16{	4
CC	GO:000567	chromatin silencing comp	2/86	13/19518	0.00145	0.004962	0.002893	BAHD1/YY1	2
CC	GO:000167	male germ cell nucleus	3/86	52/19518	0.001562	0.005206	0.003035	TAF4/TAF7/T	3
CC	GO:00017	XY body	2/86	14/19518	0.001687	0.005483	0.003197	DNMT3A/SM	2
CC	GO:00165{	RSC-type complex	2/86	15/19518	0.001941	0.005869	0.003421	SMARCB1/SM	2
CC	GO:00315{	nucleotide-activated prot	2/86	15/19518	0.001941	0.005869	0.003421	PRKAB1/PRK.	2
CC	GO:00452S	mRNA editing complex	2/86	15/19518	0.001941	0.005869	0.003421	A1CF/APOBE	2
CC	GO:00351(	PRC1 complex	2/86	16/19518	0.002212	0.006391	0.003726	CBX4/PCGF2	2
CC	GO:00715(	nBAF complex	2/86	16/19518	0.002212	0.006391	0.003726	DPF3/SMAR(	2
CC	GO:00007{	nucleosome	4/86	133/19518	0.002842	0.008033	0.004683	EPC1/EPC2/K	4
CC	GO:00358(	site of double-strand brea	3/86	78/19518	0.004952	0.013698	0.007986	RNF168/SETN	3

CC	GO:00448	DNA packaging complex	4/86	164/19518	0.005981	0.016199	0.009444	EPC1/EPC2/K	4
CC	GO:00165	RNA polymerase II, holoe	3/86	93/19518	0.008058	0.021353	0.012449	TAF4/TAF5/T.	3
CC	GO:00713	MLL1 complex	2/86	31/19518	0.008213	0.021353	0.012449	TAF4/TAF7	2
CC	GO:00446	MLL1/2 complex	2/86	32/19518	0.008735	0.022267	0.012982	TAF4/TAF7	2
CC	GO:00550	nuclear DNA-directed RN	3/86	117/19518	0.014982	0.037455	0.021836	TAF4/TAF5/T.	3
CC	GO:00004	DNA-directed RNA polym	3/86	120/19518	0.016024	0.039304	0.022914	TAF4/TAF5/T.	3
CC	GO:00308	RNA polymerase complex	3/86	124/19518	0.017475	0.042069	0.024526	TAF4/TAF5/T.	3
CC	GO:19029	protein kinase complex	3/86	142/19518	0.024888	0.058826	0.034295	CDK3/PRKAB	3
CC	GO:00364	cytoplasmic ribonucleopr	4/86	253/19518	0.025608	0.059447	0.034657	APOBEC3B/A	4
CC	GO:00357	ribonucleoprotein granule	4/86	271/19518	0.031836	0.072608	0.04233	APOBEC3B/A	4
CC	GO:00019	female pronucleus	1/86	10/19518	0.043208	0.096846	0.056461	TET3	1
MF	GO:00423	histone binding	20/87	241/18369	1.12E-19	2.38E-17	1.34E-17	BRD3/CBX4/H	20
MF	GO:01402	DNA-binding transcriptio	17/87	478/18369	8.02E-11	5.83E-09	3.27E-09	DNMT3A/FO	17
MF	GO:00616	RNA polymerase II-specifi	15/87	348/18369	8.25E-11	5.83E-09	3.27E-09	DNMT3A/FO	15
MF	GO:00169	nuclear receptor binding	10/87	138/18369	1.01E-09	5.34E-08	3.00E-08	FOXP1/KDM4	10
MF	GO:00037	transcription coactivator a	12/87	282/18369	8.64E-09	3.57E-07	2.00E-07	ARRB1/KAT6	12
MF	GO:01400	modification-dependent	10/87	175/18369	1.01E-08	3.57E-07	2.00E-07	BRCC3/BRD3	10
MF	GO:00350	methylated histone bindir	6/87	78/18369	1.85E-06	5.61E-05	3.15E-05	CBX4/KDM4A	6
MF	GO:01400	methylation-dependent p	6/87	80/18369	2.15E-06	5.70E-05	3.20E-05	CBX4/KDM4A	6
MF	GO:00037	transcription corepressor	8/87	190/18369	3.29E-06	7.75E-05	4.35E-05	CBX4/DNMT3	8
MF	GO:00314	nucleosome binding	5/87	71/18369	2.16E-05	0.000422	0.000237	RNF168/SAP3	5
MF	GO:00469	histone H3K36 methyltran	3/87	12/18369	2.19E-05	0.000422	0.000237	SETD2/SETD3	3
MF	GO:00104	histone H3 acetyltransfera	3/87	14/18369	3.60E-05	0.000602	0.000338	BRPF3/KAT6B	3
MF	GO:00168	hydrolase activity, acting	6/87	131/18369	3.69E-05	0.000602	0.000338	APOBEC3B/A	6
MF	GO:00044	histone acetyltransferase	4/87	43/18369	5.03E-05	0.000762	0.000427	BRPF3/KAT6B	4
MF	GO:00617	peptide-lysine-N-acetyltr	4/87	46/18369	6.58E-05	0.00093	0.000522	BRPF3/KAT6B	4
MF	GO:00164	acetyltransferase activity	5/87	94/18369	8.36E-05	0.001108	0.000622	BRPF3/ELP3/	5
MF	GO:00044	histone deacetylase activit	3/87	19/18369	9.41E-05	0.001166	0.000654	HDAC11/SIRT	3
MF	GO:00342	peptide N-acetyltransfera	4/87	51/18369	9.90E-05	0.001166	0.000654	BRPF3/KAT6B	4
MF	GO:00335	protein lysine deacetylase	3/87	21/18369	0.000128	0.001411	0.000792	HDAC11/SIRT	3
MF	GO:00303	nuclear receptor coactivat	4/87	55/18369	0.000133	0.001411	0.000792	NCOA2/NCO	4
MF	GO:00104	histone H4 acetyltransfera	3/87	22/18369	0.000148	0.001495	0.000839	BRPF3/KAT7/	3
MF	GO:00167	2-oxoglutarate-depender	4/87	58/18369	0.000164	0.001579	0.000886	KDM4A/KDM	4
MF	GO:00506	nuclear androgen recepto	3/87	26/18369	0.000247	0.002273	0.001275	FOXP1/KDM4	3
MF	GO:00080	N-acetyltransferase activit	4/87	67/18369	0.000287	0.002531	0.00142	BRPF3/KAT6B	4
MF	GO:00324	histone demethylase activ	3/87	30/18369	0.00038	0.003097	0.001738	KDM4A/KDM	3
MF	GO:01404	protein demethylase activ	3/87	30/18369	0.00038	0.003097	0.001738	KDM4A/KDM	3
MF	GO:00081	methyltransferase activity	6/87	208/18369	0.000462	0.003629	0.002036	DNMT3A/PR	6

MF	GO:00167	transferase activity, transfe	6/87	221/18369	0.000636	0.004815	0.002702	DNMT3A/PRI	6
MF	GO:00192	deacetylase activity	3/87	37/18369	0.00071	0.005052	0.002834	HDAC11/SIRT	3
MF	GO:00167	acyltransferase activity, tra	6/87	226/18369	0.000715	0.005052	0.002834	BRPF3/ELP3/I	6
MF	GO:00162	RNA polymerase II genera	3/87	40/18369	0.000893	0.005697	0.003197	TAF4/TAF5/T	3
MF	GO:00324	demethylase activity	3/87	40/18369	0.000893	0.005697	0.003197	KDM4A/KDM	3
MF	GO:01409	histone H3 methyltransfer	3/87	40/18369	0.000893	0.005697	0.003197	SETD2/SETD3	3
MF	GO:00512	dioxygenase activity	4/87	91/18369	0.000917	0.005697	0.003197	KDM4A/KDM	4
MF	GO:00082	protein methyltransferase	4/87	92/18369	0.000955	0.005697	0.003197	DNMT3A/SET	4
MF	GO:00518	histone H3K36 demethyla	2/87	10/18369	0.000974	0.005697	0.003197	KDM4A/KDM	2
MF	GO:00164	N-acyltransferase activity	4/87	93/18369	0.000994	0.005697	0.003197	BRPF3/KAT6E	4
MF	GO:00010	core promoter sequence-	3/87	43/18369	0.001104	0.006161	0.003457	FOXP1/SMAR	3
MF	GO:00439	histone H4K5 acetyltransf	2/87	11/18369	0.001186	0.006287	0.003527	BRPF3/KAT7	2
MF	GO:00439	histone H4K8 acetyltransf	2/87	11/18369	0.001186	0.006287	0.003527	BRPF3/KAT7	2
MF	GO:00167	acyltransferase activity	6/87	253/18369	0.001281	0.006622	0.003715	BRPF3/ELP3/I	6
MF	GO:00041	cytidine deaminase activit	2/87	12/18369	0.001419	0.006997	0.003926	APOBEC3B/A	2
MF	GO:00439	histone H4K12 acetyltrans	2/87	12/18369	0.001419	0.006997	0.003926	BRPF3/KAT7	2
MF	GO:00324	histone H3K9 demethylase	2/87	13/18369	0.001672	0.007893	0.004429	KDM4A/KDM	2
MF	GO:00012	DNA-binding transcriptio	8/87	468/18369	0.001682	0.007893	0.004429	FOXA1/GTF2I	8
MF	GO:00180	histone lysine N-methyltra	3/87	50/18369	0.001713	0.007893	0.004429	SETD2/SETD3	3
MF	GO:00012	DNA-binding transcriptio	8/87	472/18369	0.001775	0.008004	0.004491	FOXA1/GTF2I	8
MF	GO:01402	general transcription initia	3/87	54/18369	0.002138	0.009443	0.005298	TAF4/TAF5/T	3
MF	GO:00469	nuclear retinoid X recepto	2/87	15/18369	0.002237	0.009485	0.005322	NCOA6/VDR	2
MF	GO:19902	histone methyltransferase	2/87	15/18369	0.002237	0.009485	0.005322	PRDM1/PRDI	2
MF	GO:00314	chromatin DNA binding	4/87	118/18369	0.00239	0.009933	0.005573	PPARGC1A/S	4
MF	GO:00162	protein-lysine N-methyltr	3/87	60/18369	0.00289	0.011783	0.006611	SETD2/SETD3	3
MF	GO:00162	lysine N-methyltransferas	3/87	61/18369	0.003029	0.012117	0.006799	SETD2/SETD3	3
MF	GO:00012	DNA-binding transcriptio	6/87	303/18369	0.003165	0.012425	0.006971	FOXP1/FOXP	6
MF	GO:19908	promoter-specific chroma	3/87	63/18369	0.00332	0.012797	0.00718	IKZF3/PCGF2	3
MF	GO:00012	DNA-binding transcriptio	6/87	308/18369	0.00343	0.012983	0.007285	FOXP1/FOXP	6
MF	GO:00428	histone H3K4 methyltrans	2/87	19/18369	0.003598	0.013257	0.007438	SETD3/SETM	2
MF	GO:00420	histone methyltransferase	3/87	65/18369	0.003627	0.013257	0.007438	SETD2/SETD3	3
MF	GO:00168	hydrolase activity, acting	3/87	68/18369	0.004119	0.014801	0.008305	HDAC11/SIRT	3
MF	GO:00351	histone kinase activity	2/87	21/18369	0.004392	0.015519	0.008707	JAK2/PRKCB	2
MF	GO:00705	lysine-acetylated histone	2/87	23/18369	0.005259	0.017983	0.01009	BRD3/TRIM24	2
MF	GO:01400	acetylation-dependent pr	2/87	23/18369	0.005259	0.017983	0.01009	BRD3/TRIM24	2
MF	GO:00429	nuclear retinoic acid recep	2/87	24/18369	0.00572	0.019247	0.010799	NCOA6/VDR	2
MF	GO:00197	ubiquitin-like protein tran	7/87	467/18369	0.006681	0.022129	0.012416	CBX4/RAG1/I	7
MF	GO:00087	S-adenosylmethionine-de	4/87	162/18369	0.007359	0.024003	0.013467	DNMT3A/SET	4

MF	GO:00469(	nuclear thyroid hormone r	2/87	29/18369	0.008286	0.026615	0.014933	NCOA6/TAF7	2
MF	GO:00167(	oxidoreductase activity, ac	4/87	177/18369	0.009975	0.03127	0.017545	KDM4A/KDM	4
MF	GO:00192:	deaminase activity	2/87	32/18369	0.01003	0.03127	0.017545	APOBEC3B/A	2
MF	GO:00616:	ubiquitin conjugating enz	2/87	33/18369	0.010644	0.032705	0.01835	UBE2A/UBE2I	2
MF	GO:00081	N-methyltransferase activ	3/87	99/18369	0.011609	0.035158	0.019726	SETD2/SETD3	3
MF	GO:00168	hydrolase activity, acting c	2/87	35/18369	0.011922	0.035598	0.019973	APOBEC3B/A	2
MF	GO:00045	DNA endonuclease activit	2/87	36/18369	0.012585	0.037055	0.020791	RAG1/SETMA	2
MF	GO:00616	ubiquitin-like protein con	2/87	37/18369	0.013263	0.038518	0.021612	UBE2A/UBE2I	2
MF	GO:00316	ubiquitin protein ligase bi	5/87	301/18369	0.014175	0.040609	0.022785	ARRB1/CUL4I	5
MF	GO:00303:	nuclear estrogen receptor	2/87	39/18369	0.014667	0.041459	0.023262	NCOA6/PADI	2
MF	GO:00046	protein serine/threonine k	6/87	421/18369	0.014877	0.041499	0.023284	ATM/CDK3/P	6
MF	GO:00314	nucleosomal DNA binding	2/87	41/18369	0.016132	0.044416	0.024921	SMARCB1/SM	2
MF	GO:00321	ubiquitin-like protein binc	3/87	114/18369	0.016921	0.04599	0.025804	CBX4/RNF168	3
MF	GO:01010(	deubiquitinase activity	3/87	115/18369	0.017316	0.046468	0.026072	BRCC3/MYSM	3
MF	GO:00048	ubiquitin-protein transfera	6/87	438/18369	0.017737	0.047003	0.026372	RAG1/RNF16	6
MF	GO:00443	ubiquitin-like protein liga	5/87	320/18369	0.018	0.04711	0.026432	ARRB1/CUL4I	5
MF	GO:00045	endonuclease activity	3/87	122/18369	0.020225	0.052289	0.029338	PIWIL4/RAG1	3
MF	GO:00428	histone deacetylase bindir	3/87	126/18369	0.022001	0.056194	0.031529	IKZF3/SATB2/	3
MF	GO:00469	protein heterodimerizatio	5/87	343/18369	0.023475	0.059248	0.033242	CHRAC1/IKZI	5
MF	GO:00197	ubiquitin-like protein pep	3/87	130/18369	0.023859	0.059508	0.033388	BRCC3/MYSM	3
MF	GO:00315	polyubiquitin modificatior	2/87	55/18369	0.027998	0.069018	0.038724	BRCC3/RNF1	2
MF	GO:01400	catalytic activity, acting or	4/87	245/18369	0.029078	0.070856	0.039755	CHRAC1/DNI	4
MF	GO:00045:	deoxyribonuclease activity	2/87	57/18369	0.029909	0.072053	0.040427	RAG1/SETMA	2
MF	GO:00020:	p53 binding	2/87	67/18369	0.040199	0.095755	0.053726	SMARCB1/TR	2
MF	GO:00036	damaged DNA binding	2/87	71/18369	0.044639	0.099312	0.055721	CUL4B/SIRT6	2
MF	GO:00000	single-stranded DNA end	1/87	10/18369	0.046377	0.099312	0.055721	SETMAR	1
MF	GO:00011	RNA polymerase I transcri	1/87	10/18369	0.046377	0.099312	0.055721	SMARCB1	1
MF	GO:00011	RNA polymerase I core pr	1/87	10/18369	0.046377	0.099312	0.055721	SMARCB1	1
MF	GO:00081	S-methyltransferase activi	1/87	10/18369	0.046377	0.099312	0.055721	DNMT3A	1
MF	GO:00163(	1-phosphatidylinositol-3-	1/87	10/18369	0.046377	0.099312	0.055721	ATM	1
MF	GO:00168	hydrolase activity, acting c	1/87	10/18369	0.046377	0.099312	0.055721	PADI2	1
MF	GO:00309	Tat protein binding	1/87	10/18369	0.046377	0.099312	0.055721	SMARCB1	1
MF	GO:00320	bile acid binding	1/87	10/18369	0.046377	0.099312	0.055721	VDR	1
MF	GO:01404	chromatin-protein adaptc	1/87	10/18369	0.046377	0.099312	0.055721	EPC1	1

table S3

category	subcategor	ID	Descriptior	GeneRatio	BgRatio	pvalue	p.adjust	qvalue	geneID	Count
Genetic Inf	Chromosor	hsa03082	ATP-deper	7/44	117/8868	1.49E-06	0.000198	0.000167	CHRAC1/D	7
Genetic Inf	Chromosor	hsa03083	Polycomb	5/44	83/8868	5.19E-05	0.002746	0.002325	CBX4/PCGI	5
Genetic Inf	Transcripti	hsa03022	Basal trans	4/44	44/8868	6.19E-05	0.002746	0.002325	GTF2I/TAF4	4
Organisma	Environme	hsa04714	Thermoger	7/44	235/8868	0.000139	0.004632	0.003923	DPF3/PPAF	7
Organisma	Endocrine	hsa04920	Adipocytol	4/44	70/8868	0.000381	0.010132	0.00858	JAK2/PPAR	4
Organisma	Environme	hsa04710	Circadian r	3/44	34/8868	0.000613	0.013579	0.011499	NPAS2/PRI	3
Human Dis	Endocrine	hsa04931	Insulin resis	4/44	109/8868	0.002009	0.038163	0.032319	PPARGC1A	4
Genetic Inf	Informatior	hsa03250	Viral life cy	3/44	64/8868	0.003846	0.054884	0.046479	APOBEC3B	3
Human Dis	Infectious c	hsa05170	Human imı	5/44	213/8868	0.003866	0.054884	0.046479	APOBEC3B	5
Environme	Signal tran	hsa04068	FoxO signa	4/44	133/8868	0.004127	0.054884	0.046479	ATM/PRKA	4
Human Dis	Cancer: spe	hsa05225	Hepatocell	4/44	170/8868	0.009745	0.109909	0.093077	DPF3/PRKC	4
Organisma	Aging	hsa04211	Longevity r	3/44	90/8868	0.009917	0.109909	0.093077	PPARGC1A	3
Organisma	Endocrine	hsa04922	Glucagon s	3/44	107/8868	0.015807	0.161718	0.136951	PPARGC1A	3
Genetic Inf	Replication	hsa03440	Homologo	2/44	41/8868	0.017453	0.165807	0.140414	ATM/BRCC	2
Organisma	Endocrine	hsa04928	Parathyroic	3/44	115/8868	0.019133	0.169646	0.143665	ARRB1/PRK	3
Human Dis	Cancer: ove	hsa05207	Chemical c	4/44	215/8868	0.021395	0.174767	0.148002	ARRB1/JAK	4
Environme	Signal tran	hsa04152	AMPK sign	3/44	122/8868	0.022339	0.174767	0.148002	PPARGC1A	3
Organisma	Excretory s	hsa04961	Endocrine	2/44	53/8868	0.028253	0.20684	0.175163	PRKCB/VD	2
Organisma	Endocrine	hsa04910	Insulin sigr	3/44	138/8868	0.030705	0.20684	0.175163	PPARGC1A	3
Environme	Signal tran	hsa04371	Apelin sign	3/44	140/8868	0.031852	0.20684	0.175163	PPARGC1A	3
Genetic Inf	Folding, so	hsa04120	Ubiquitin n	3/44	142/8868	0.033022	0.20684	0.175163	CUL4B/UBI	3
Human Dis	Endocrine	hsa04936	Alcoholic li	3/44	144/8868	0.034214	0.20684	0.175163	PPARGC1A	3
Organisma	Aging	hsa04213	Longevity r	2/44	62/8868	0.037702	0.210588	0.178337	PRKAB1/PF	2
Metabolisn	Amino acic	hsa00310	Lysine deg	2/44	63/8868	0.038817	0.210588	0.178337	SETD2/SET	2
Organisma	Endocrine	hsa04929	GnRH secre	2/44	65/8868	0.041086	0.210588	0.178337	ARRB1/PRK	2
Organisma	Endocrine	hsa04921	Oxytocin si	3/44	155/8868	0.041168	0.210588	0.178337	PRKAB1/PF	3
